# Machine learning for cognitive behavioral analysis: datasets, methods, paradigms, and research directions

**DOI:** 10.1186/s40708-023-00196-6

**Published:** 2023-07-31

**Authors:** Priya Bhatt, Amanrose Sethi, Vaibhav Tasgaonkar, Jugal Shroff, Isha Pendharkar, Aditya Desai, Pratyush Sinha, Aditya Deshpande, Gargi Joshi, Anil Rahate, Priyanka Jain, Rahee Walambe, Ketan Kotecha, N. K. Jain

**Affiliations:** 1 Symbiosis Institute of Technology, Symbiosis International Deemed University, Pune, India; 2 Symbiosis Centre for Applied Artificial Intelligence, Symbiosis International Deemed University, Pune, India; 3 Centre for Development of Advanced Computing (C-DAC), Delhi, India; 4 UCSI University, Kuala Lumpur, Malaysia

**Keywords:** Cognitive behavior analysis, Multimodal AI, Multimodal data fusion, Affective computing, Brain-inspired AI, Deception detection

## Abstract

Human behaviour reflects cognitive abilities. Human cognition is fundamentally linked to the different experiences or characteristics of consciousness/emotions, such as joy, grief, anger, etc., which assists in effective communication with others. Detection and differentiation between thoughts, feelings, and behaviours are paramount in learning to control our emotions and respond more effectively in stressful circumstances. The ability to perceive, analyse, process, interpret, remember, and retrieve information while making judgments to respond correctly is referred to as Cognitive Behavior. After making a significant mark in emotion analysis, deception detection is one of the key areas to connect human behaviour, mainly in the forensic domain. Detection of lies, deception, malicious intent, abnormal behaviour, emotions, stress, etc., have significant roles in advanced stages of behavioral science. Artificial Intelligence and Machine learning (AI/ML) has helped a great deal in pattern recognition, data extraction and analysis, and interpretations. The goal of using AI and ML in behavioral sciences is to infer human behaviour, mainly for mental health or forensic investigations. The presented work provides an extensive review of the research on cognitive behaviour analysis. A parametric study is presented based on different physical characteristics, emotional behaviours, data collection sensing mechanisms, unimodal and multimodal datasets, modelling AI/ML methods, challenges, and future research directions.

## Introduction

**Fig. 1:**
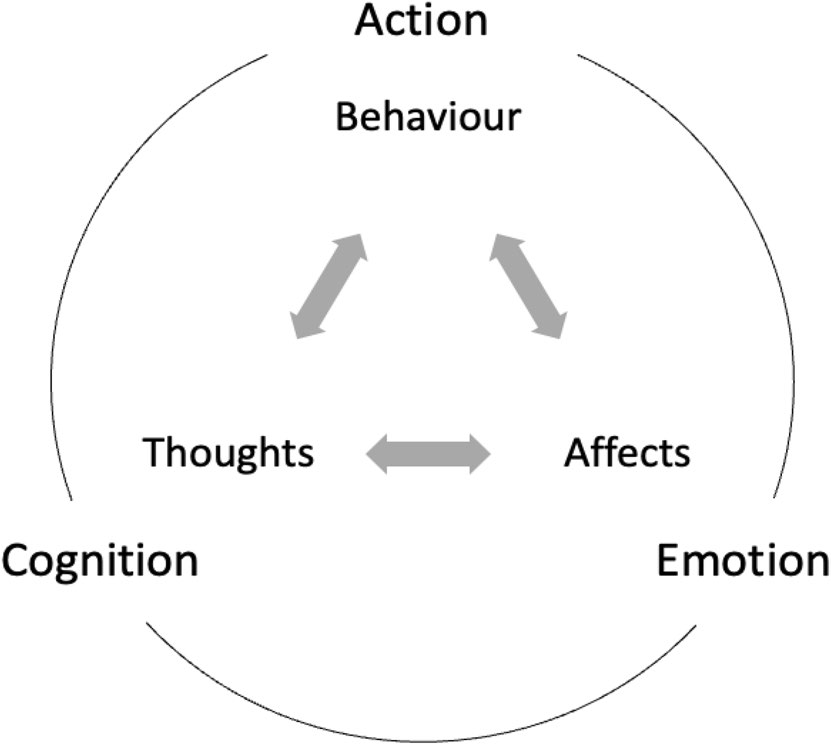
Interaction between Cognition, Emotion, and Action

Three factors interact intricately to form human behaviour: actions, cognition, and emotions. These factors have prominent roles in observing abnormalities or anomalies in behaviour. Actions are everything that can be observed and measured through sensors. Cognitions are verbal and non-verbal thoughts and mental images. They also consist of skills, knowledge, and experience a person gains. An emotion is a temporary mental state characterized by intense cognitive activity and a feeling that is not considered to result from knowledge or reasoning [62]. Typically, there is a scale for this, with positive (pleasurable) and negative values (unpleasant) [62]. The interaction among cognition, emotion, and action is shown in Fig. 1. Through this review, we aim to highlight Artificial Intelligence algorithms' developments in trying to quantify and perceive human behaviour.

The authors in [61] reviewed personal and social factors influencing human behaviour. Personal factors that influence emotions include experiences from childhood, education and knowledge, personality and self-concept, sense of control, values, political and world views, goals, perceived responsibility, cognitive biases, emotional attachment to a specific place, age, gender, and chosen activities. Social factors that play a role include religion, differences between urban and rural areas, societal norms, social class, proximity to areas of environmental concern, and cultural and ethnic variations.

The principle of human psychology is always developed from inputs received by behaviour influenced by emotion. Emotions such as happiness, fear, sadness, wrath, shock, thrill, guilt, regret, hatred, and intrigue are distinct from behaviour but play an important role in driving some actions. Human emotion recognition is of utmost importance for understanding human behaviour laws, the study and the practice of sociology, management, and economics. Various popular psychological therapies, like Cognitive Behavioral Therapy (CBT), could be utilized to treat human behaviour and emotions. CBT is also a well-researched psychological strategy for treating people with symptoms that have no known medical cause [77].

### Cognitive behavior analysis

In philosophy and political psychology, "cognition" typically refers to conscious and intentional processes involved in thought and knowledge. This understanding of cognition aligns with the origins of cognitive neuroscience within cognitive psychology, which focuses on investigating mental processes such as perception, attention, memory, language [69] learning, reasoning, judgment, and higher-order thinking that can be deliberately controlled [97]. On the other hand, emotion is characterized as a sudden, temporary state of agitation or disturbance caused by an intense experience, such as fear, surprise, or joy, or as a mental state or feeling, such as pain, desire, or hope, that is distinct from cognitive processes or intentional actions [98]. Though Emotion and cognition have traditionally been considered distinct systems, the relationship between cognition and emotion is more interdependent than separate, according to recent studies in the cognitive and neurobiological sciences [82]. Researchers increasingly realise that cognition and emotion processes interact and that their neurological mechanisms are integrated within the brain to influence behaviour mutually. The general developing theory also holds that our behaviours, emotions, and actions all impact how we feel, act, and think in a self-reinforcing feedback system. As cognition, emotion, and behaviour are interdependent, cognitive decline can lead to major setbacks and psychological problems. When individuals seek treatment for such problems as depression and other mental disorders impairing their everyday physical and social functioning, cognitive behavioral therapy (CBT) is helpful. Various issues, including anxiety, emotional problems, alcoholism, marriage problems, poor self-esteem, and serious mental diseases, have been successfully treated with CBT. CBT looks at how thoughts, emotions, and behaviours are connected. It is a structured, short-term, directive approach that aims to help patients develop more adaptive thoughts and behaviours and reduce distress [84]. Moreover, CBT involves Cognitive Behavioral Analysis, where it can be understood or detected whether a person is lying, performing deceptive actions, behaving abnormally, stressed, or having malicious intentions.

### Role of Artificial Intelligence-Generated Content (AIGC) in Cognitive Behaviour Analysis

AIGC leverages artificial intelligence to generate content automatically or assist in content generation based on user-provided keywords or requirements. The advancement of large model algorithms has significantly enhanced the capabilities of AIGC, making it a promising tool for generating content and enhancing convenience in our daily lives [99, 100]. AIGC can play a significant role in cognitive behaviour analysis by providing valuable insights and support such as in data analysis and pattern recognition, automated data collection, natural language processing, Personalised interventions and recommendations, real-time monitoring and feedback, virtual assistants and chatbots, Various frameworks like Generative Adversarial Networks (GAN), Variational Autoencoder (VAE), Dual-Variational Autoencoder (D-VAE), Natural Language Processing, etc. help in the analysis of cognitive behaviour or performing brain analysis aligned work is presented in [101–107].

### Need and motivation for deception detection

Integrating Artificial Intelligence with the accessibility of vast amounts of data and improved computational capabilities are driving advancements in fields such as genetics, climate research, and astronomy. AI aims to mimic the human brain, but understanding human behaviour is difficult as behavioral science is a complex subject, to begin with. AI in behavioral sciences is still evolving and is typically not regarded as a primary evaluation and interpretation method.

In this study, we have summarized the developments made by AI in Cognitive Behavior Analysis. In the rapidly expanding field of mental health research, AI is a method that seeks to examine the issue from a statistical perspective to maximise prediction. Due to its data-driven and multivariate nature, machine learning is better suited to handle complicated and varied issues like forecasting who would experience a mental illness or relapse, predicting emotions, detecting stress, detecting lies, identifying intent, etc. Initially, unimodal channels like audio, images, text, and physiological channels such as ECG, EEG, GSR, BVP, etc., have been used to predict and classify cognitive behaviour along with AI/ML models. However, [68] introduced a multimodal foundation model pre-trained on 15 million image-text pairs and demonstrated strong multimodal understanding and generalisation abilities in several cognitive downstream tasks. Furthermore, multimodal-trained visual and lingual encoders are more brain-like than unimodal ones from the perspective of neural encoding. In light of this, multimodal foundation models are better resources for neuroscientists to use when examining the multimodal signal processing systems in the human brain [68].

Also, the lack of consolidated information on the use of AI/ML for all psychological aspects, such as lying, deception, deviant behaviour, stress, and emotion, is the driving force for this review. This review would be useful for learning about different datasets and techniques used for these diverse cognitive processes.

### Research questions

Focusing on Cognitive-behavioral approaches, this paper is centric on abnormality detection in human behaviour. Cognitive Behavior includes emotions, stress, lie, deception, deviant behaviour, etc. Cognitive Behavior Analysis uses machine learning to understand sensing mechanisms, machine learning methods, available datasets, data preprocessing and feature extraction approaches, and model development pipelines for each CBA behaviour. This review addresses the following questions:

1. What are the existing challenges in traditional cognitive behaviour analysis?
2. What unimodal and multimodal datasets are available for cognitive behaviour analysis using AI/ML?
3. What methods are to be applied while extracting features from different modalities?
4. Is there a generalised pipeline in place while performing different CBA detections?
5. What are the challenges and future research directions in applying AI/ML in CBA?

### Novelty and contribution

Though the works on emotion detection have been reported by many researchers recently, malicious behaviour detection is one research area where lots of effort is put upon. Several recent studies have reported various aspects of human behaviour analysis using AI. In the paper [59], the author reviewed several studies about stress detection using machine learning techniques based on physiological sensors. In research [60], the author has reviewed studies showing how machine learning can help predict who can benefit from CBT. In the publication [39], the author has depicted how the Facial Action Coding System (FACS) detects deception in videos.

Similarly, in reported works [14] and [15], AI/ML methods are shown to detect abnormal behaviour in humans. These previous studies mainly focus on one or two behavioral analyses, for example, stress detection, abnormal behaviour, or deception detection. We covered multiple behavioral analyses in a single study, namely, emotions, stress, abnormal behaviour, and deception detection, through reviewing 100 papers that presented works on Cognitive Behavioral Analysis. We collated all the essential details into three categories: Lie/Deception detection, stress/emotion detection, and abnormal behaviour detection. We also presented a detailed review of the unimodal and multimodal datasets and the methodologies used in each category. In this review, we have principally considered reviewing various machine learning algorithms like Random Forest Classifier, Support Vector Classifier (SVC), AdaBoost (AB), etc., and Deep learning models like Artificial neural network (ANN), Recurrent Neural Networks (RNN), Convolutional Neural Network (CNN), etc. to classify human emotions on unimodal and multimodal datasets.

### Organisation of the paper

This paper is structured in the following way:

- Sect. 2 explains the different types of behaviours discussed in the literature.
- Sect. 3 describes the datasets that are available for these behaviours.
- The AI and ML techniques and methodologies used are discussed in Sect. 4.
- Sect. 5 includes a discussion of the challenges,
- And the future potential of this research is discussed in Sect. 6.

## Types of behavioral analysis

**Fig. 2:**
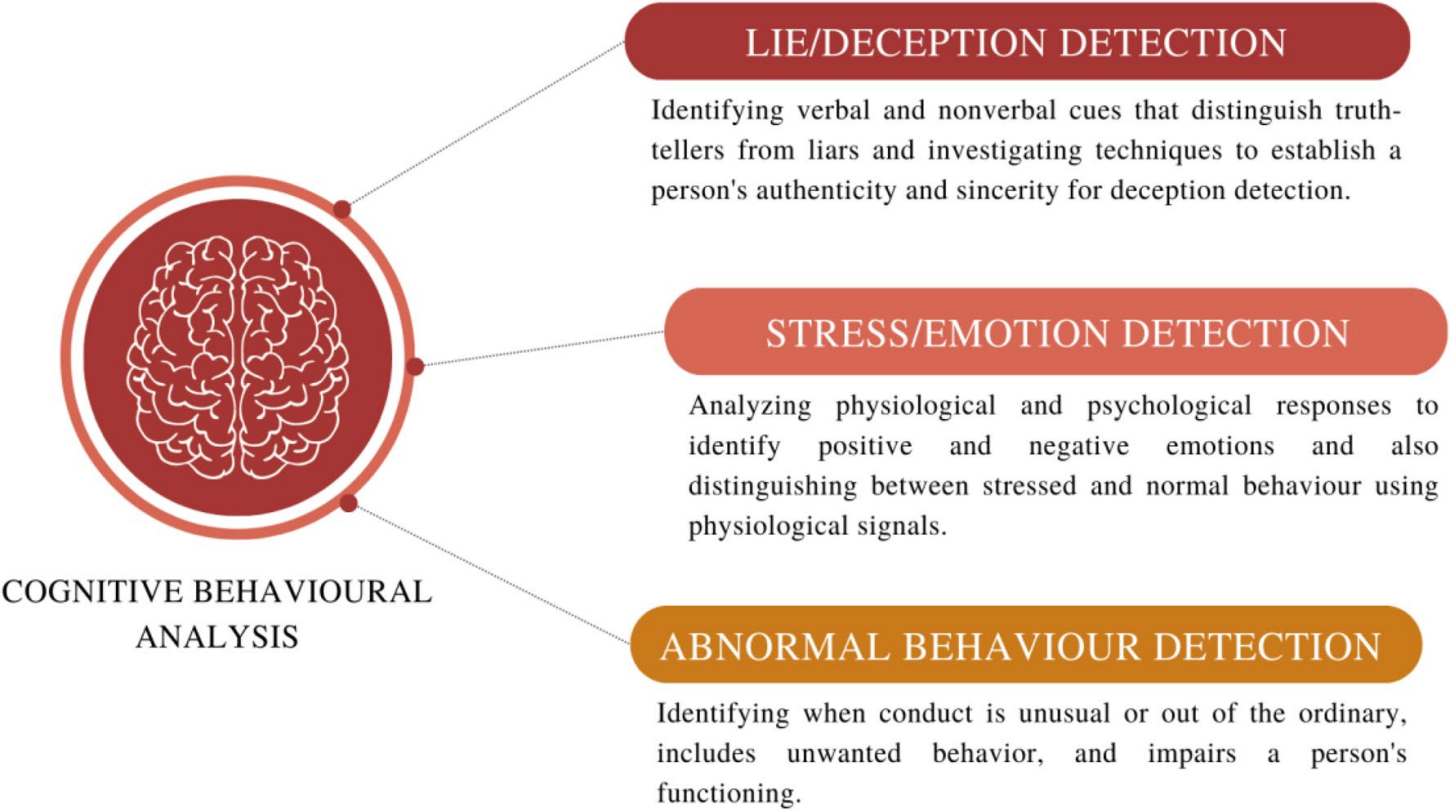
Broad classification of cognitive behavioral analysis

The research work [79] proposes that intrusive thoughts should be viewed as cognitive stimuli rather than responses after thoroughly examining cognitive and behavioral models. These stimuli are often linked to negative automatic thoughts about personal or social responsibility or blame. Cognitive Behavioral Analysis is required to understand the occurrences of such cognitive reactions. Here, a review of cognitive behavioral analysis of psychological problems like negative thoughts, lies, stress, and abnormal behaviour is presented. Hence, this study has mainly divided Behavioral Analysis into three types, as shown in Fig. 2:

- Lie/Deception Detection,
- Stress/Emotion Detection,
- Abnormal Behavior Detection.

The datasets and algorithms are classified as unimodal and multimodal, considering implementations.

### Lie/deception detection

One of the most frequent and harmful behaviours people commit, lying, is worth reflecting on. The goal of lie detection is to identify verbal and nonverbal cues that distinguish truth-tellers from liars. Detecting the difference between liars and truth-tellers has been a topic of interest throughout history. Questioning strategies are mainly used in lie or deception detection to determine truth and untruth in responses [70]. However, human beings are habitual in observing and understanding cues by viewing. Hence, the most transparent approach in every study was to record videos or take pictures. In research [1], a person's ability to judge deception was examined by viewing video clips. Five independent experiments were conducted where children and adults were recorded telling lies/truth through ‘yes’/‘no’ and open-ended questions. These video clips were then classified as liars and truth-tellers. The study gave unreliable results and suggested using more than video clips for deception detection. The various methods through which deception can be scientifically detected [2] are as follows:

- Based on behavioral and non-verbal cues
  o Strategic Use of Evidence [3]
  p Verifiability Approach [4]
  q Cognitive Load Approaches [5]
- Verbal Detection Methods
  o Reality Monitoring [6]
  p Criteria-based Content Analysis (CBCA) [7]
  q Lie Detection Tests (Popularly known as polygraph tests) [85] – Comparison Question Test (CQT) [93] and Concealed Information Test (CIT) [94]

High-stakes lies are frequently told by suspects in police interrogation of criminals, terrorists, traffickers at airports, dishonest politicians when speaking with untrustworthy journalists, and family relationships like adultery behaviours [5]. Deceptive actions might be as basic as harmless lies or as serious as threats. Since dishonesty permeates nearly every human encounter and can have expensive repercussions, deception detection has drawn increased interest from various research communities, including computer vision, psychology, and language processing [75]. Due to the serious security crises that have happened recently, global interest has arisen in finding liars. Airports are one location where it is crucial to spot dishonesty. Terrorists can lie to interviewers at customs and borders and hide vital information that could endanger people's lives. The legal system offers another illustration. Every day, thousands of cases occur where juries must make important judgments based on facts and their human judgment that may significantly impact the lives of suspects and victims.

Applications, including security, business, and investigative techniques, sparked interest in research across other disciplines. Most current approaches rely on polygraph tests that gather physiological data, including skin conductance, skin temperature, respiration rate, and heart rate [75]. This strategy has often been shown to accuse the innocent and release the guilty. Polygraph tests have frequently been proved to be erroneous since human experts must make such decisions prone to bias and inaccuracy. Additionally, with the right training, suspects might pretend innocent by employing specialized techniques like lying when answering pre-test questions, tensing their muscles, or biting their tongues. Alternative strategies were developed using Artificial Intelligence to increase the dependability of deception detection systems since detecting dishonesty has expanded to various applications such as social media, interviews, online transactions, and deception in everyday life [75].

### Stress/emotion detection

Emotion is a mental and physiological process triggered by the awareness or unawareness of an object or situation [96]. It is frequently linked to mood, temperament, character, inclination, and desire. Emotions play a significant part in human communication and can be represented verbally through emotional language or nonverbally through gestures, facial expressions, and voice intonation. Emotion Detection analyses physiological and psychological responses to identify positive and negative emotions. Negative emotions could also lead us to stress.

Stress is an intensified psycho-physiological state of the body that develops in reaction to a demanding situation or challenging occurrence [59]. Stressors are the variables in the environment that cause stress. Prolonged exposure to multiple stressors can negatively impact an individual's mental and physical health and lead to chronic health issues. It is crucial to detect stress-related problems early on to prevent them, and this can only be accomplished through continuous monitoring of stress levels. The feeling of stress is frequently accompanied by visual clues [8]. Self-directed behaviours such as lip-biting, face-touching, and scratching are among them. In [9], the importance of non-verbal behaviour in stress detection has been presented. They tried to investigate whether stress-related behaviours known as displacements accurately predict stress in people. Examples of actions considered displacement behaviours include grooming, touching one's face, biting or licking lips, scratching, yawning, fidgeting, twisting the mouth, and the sum of the duration of these actions to obtain a displacement score. To do this, raters were shown recordings of people (N = 31) completing a stressful job (N = 133). Self-reported stress and self-directed displacement behaviours were linked to raters' stress ratings. As a result, these actions might be seen as communicative and can give others accurate information. There may be an advantage and an adaptive role to showing stress, as seen by raters' higher ratings of likeability for people who exhibit more nonverbal stress behaviour. The ability of the raters to identify stress from nonverbal cues varied as well. Findings reveal that the quantity of social connections people reported having was related to how well they could identify stress. Individuals who said the fewest network connections were the most and least accurate showed that this association was non linear [9]​.

### Malicious intent detection

Having or displaying the desire to harm someone is known as having malicious intent, which can be given to, marked by, or resulting from malice. With the sudden rise in social media, micro-blogging sites like Twitter have already started detecting, censoring, and blocking people with "hateful" behaviour. In [10], a model is presented to detect criminal acts on Social Media Websites with 8,835,290 tweets as a corpus. On the other hand, the [11] presents non-verbal cues of malicious intent. In this scenario, a trainee monitors a checkpoint and questions individuals about their identities and reasons for entering a restricted area. While most interactions are typical and without incident, there may be instances where the trainee is required to ask additional questions and make quick decisions during a specific situation. In these unique situations, people must use their understanding of social interactions to respond appropriately and make the right decisions. The research program outlined in [11] aims to create emotional states for intelligent agents, emotional behaviours that indicate cues for anger, nervousness, and deception, and a comprehensive questioning training environment that facilitates the development of decision-making based on judgment [11]. Malicious intents can be detected through speech, video, and audio. It is also possible to use physiological sensors, such as functional magnetic resonance imaging (fMRI) or electroencephalography (EEG), to identify patterns of brain activity that may indicate malicious intent. However, this technology is still developing and needs to be more reliable to identify malicious intent in real-world settings. Additionally, interpreting brain activity patterns is a complex task requiring neuroscience and machine learning expertise.

### Abnormal behavior detection

When behaviour is unexpected or out of the ordinary, including undesirable behaviour and poor functioning, any behaviour that deviates from accepted societal, cultural, and ethical norms is considered abnormal. It is usual for people with mental illnesses to behave abnormally or violently. When people with mental illnesses behave abnormally in public settings, they risk hurting themselves and other people's bodies and minds. As a result, it's important to use visual surveillance devices to keep an eye on their activities [12]. AI has proved significant with several techniques like YOLOv3 combined with the K-Means algorithm, GIoUloss, focal loss, and Darknet32, which has yielded promising results in detecting such abnormal behaviours [13]. The author [83] presented their work using activity recognition to detect early indicators of motion and cognitive impairment or abnormal activities through tracking motion and cognitive abilities. Several methods, including decision trees, k-Nearest Neighbors, Multilayer Perceptron Neural Networks, SVMs, Fuzzy Logic, Regression models, Markov models, and classifier ensembles like Boosting and bagging have been used to solve the problem [83].

## Cognitive behavior analysis datasets

This section discusses various unimodal and multimodal datasets used for Lie/Deception Detection, Stress/Emotion Detection, and Abnormal Behavior Detection. In certain conditions, these datasets describe data captured in various formats like audio, video, text, physiological signals, etc. We also discussed the data acquisition methods commonly used. Table 1 provides an overview of the datasets:

**Table 1:** Cognitive Behavior Analysis Datasets and Data Acquisition Methods

Dataset Name	Data Modalities	Dataset Type	Cognitive Behavior Analysis Task	Description
Miami University Deception Detection Database (MU3D) [15]	Videos	Multimodal	Lie/Deception Detection	The dataset comprises 320 videos featuring individuals providing both truthful and deceptive statements. It is a multimodal dataset encompassing various data modalities, including audio and video [15]. This dataset contains 320 films of individuals telling lies and stating the truth
Silesian Deception Database [16]	Videos	Multimodal	Lie/Deception Detection	The dataset consists of 101 high-speed camera video recordings of subjects captured at a resolution of 640*480 and a frame rate of 100 frames per second. Within the database, over 1.1 million coded frames serve as the ground truth for detecting deception cues on the subject's face during truthful and deceptive statements. The videos of subjects were captured with a 640*480 resolution and 100 frames per second
ReLiDDB (ReGIM-Lab Lie Detection Database) [17]	Speech Signals	Unimodal	Lie/Deception Detection	The dataset consists of recordings of false and true declarations captured in various indoor and outdoor settings. It includes 40 subjects' speech signals, presenting hypothetical scenarios for preliminary investigation. The dataset contains approximately 37% samples of false declarations and 68% samples of true declarations. A dataset containing speech signals that can be used for preliminary investigation
Deception Detection and Physiological Monitoring (DDPM) [58]	Thermal video frames, text (annotations), audio, and pulse oximeter for 70 subjects over 13 h	Multimodal	Lie/Deception Detection	The database encompasses approximately 13 h of recordings from 70 subjects, comprising over 8 million video frames captured in visible-light, near-infrared, and thermal spectra. Additionally, the database includes relevant metadata, audio data, and pulse oximeter data [58]. The interviewee's data, including RGB, near-infrared, long-wave infrared recordings, cardiac pulse, blood oxygenation, and audio information, were collected and annotated for further analysis [58]. A situation in which the interviewee tries to trick the interviewer by giving certain answers
SJTU Emotion EEG data set (SEED) [21]	EEG Signals	Unimodal	Stress/Emotion Detection	The SEED dataset, or the SJTU Emotion EEG Dataset [21], consists of three-class emotional EEG data obtained from 15 individuals. During the data collection, participants were exposed to emotional film clips representing positive, negative, and neutral emotions [21].An EEG dataset acquired from 15 subjects
EEG data set for genuine & acted emotional expressions [52]	EEG Signals	Unimodal	Stress/Emotion Detection	The dataset involves classifying emotions into genuine, neutral, or simulated categories. During the data collection process, participants wore an EEG headset while being presented with photos or movie clips displayed on a computer monitor [52]. The participants' emotions experienced fluctuations in response to the visual stimuli, reflected in the captured EEG data [52]. EEG recordings of subjects with genuine and fake emotional expressions
A Database for Emotion Analysis using Physiological Signals (DEAP) [20]	EEG signals, peripheral physiological signals, and multimedia content analysis	Multimodal	Stress/Emotion Detection	In this dataset, 32 individuals' EEG and peripheral physiological data were monitored while they watched 40 one-minute-long music video snippets. Each film was scored by participants based on its arousal, valence, like/dislike, dominance, and familiarity levels. Frontal face footage was also taken for 22 of the 32 participants [20].EEG recordings and peripheral physiological signals of 32 subjects as each watched 40 one-minute-long excerpts of music videos
SWELL_KW dataset [22, 46]	Computer logs, facial expressions from video recording, Body postures and HRV	Multimodal	Stress/Emotion Detection	This dataset contains readings from 25 participants, subjected to neutral interruptions and pressure conditions for 3 h each [22]. The data collected are computer logs, facial expressions from video recording, body postures using the Kinect 3D sensor, the ECG sensor, and the body sensors for skin conductance level. A dataset that contains readings from 25 participants who were subjected to neutral, interruptions, and pressure conditions for a total of 3 h each
Physical Activity and Stress (PASS) dataset [23]	ECG, EDA, respiration, temperature	Multimodal	Stress/Emotion Detection	This dataset consists of the experimental procedure employed and descriptive statistics of the participants' neurophysiological signals captured under various circumstances. Tasks of varying stress levels were asked to be performed by participants
Continuous stress detection on nurses in a hospital [24]	EDA, ECG, accelerometer data, temperature	Multimodal	Stress/Emotion Detection	This dataset provides physiological stress indicators for nurses working in real hospital environments during the COVID-19 pandemic. It was created primarily to conduct research on stress in the workplace setting and was collected using data streams from Empatica E4 devices [24]. Physiological data were monitored. Survey was filled out by nurses periodically regarding the aspects that contributed to stress
PURE [25]	Video, pulse rate, SpO2 readings	Multimodal	Stress/Emotion Detection	Ten subjects were asked to perform different head-head motions. This benchmark dataset focuses on how much the head moves during the measurement was introduced [25] motions
COFACE [26]	Videos, physiological signals (contact photoplethysmography and respiration)	Multimodal	Stress/Emotion Detection	This dataset includes 160 movies and physiological information collected from 40 healthy adults over several days. The group was composed of 70% men and 30% women. Participants were recorded for one minute using a standard webcam, while their physiological data were recorded using a Blood-Volume Pulse sensor and a respiration belt [26].Data collected from 40 subjects over several days for realistic conditions
MAUS [27]	ECG, PPG, GSR signals	Multimodal	Stress/Emotion Detection	This dataset includes data collected from 22 healthy graduate students who were given guidelines and signed a consent form before the test [27]. The study participants' age had an average of 23 years and a standard deviation of 1.7. The mental workload was checked using wearable sensors
VIPL-HR [28]	Visible light videos, Near infrared videos	Multimodal	Stress/Emotion Detection	This dataset consists of 2,378 visible light videos (VIS) and 752 near-infrared (NIR) videos, capturing 107 subjects. The VIPL-HR database encompasses diverse variations, including head movements, illumination variations, and changes in acquisition devices [28]. Remote heart rate estimation was done using a face Videos
(Multimodal Sentiment Analysis) – Stress (MuSe-Stress) dataset [29]	Text, audio, video, and physiological data like skin temperature, skin conductance, breathing rate, and heart rate	Multimodal	Stress/Emotion Detection	This dataset consists of stressed emotions that consist of recordings of 28 college students from the University of Michigan, nine females and 19 males, in two sessions: one during which an external stressor (the University of Michigan's final exam period) was present, and the other session during which the stressor was absent. Each recording lasts about 45 min in total. Each individual is exposed to various emotional stimuli, including brief movies and questions that evoke strong emotions [29]. Three separate datasets are used to analyse sentiments and detect emotions and humour
Koko website [30]	Text (corpus)	Unimodal	Abnormal Behavior Detection	The corpus contained 500,000 written posts and was annotated into three classes, thinking errors, emotions, and situations [30] and was annotated into three classes, thinking errors, emotions, and situations
Abnormal crowd behaviour dataset [31]	Video	Multimodal	Abnormal Behavior Detection	This dataset is a collection of normal and abnormal crowd recordings. The collection consists of films from 11 different escape event scenarios shot in 3 indoor and outdoor settings. Each video starts with a segment on regular behaviour and concludes with segments on deviant conduct [31].Computer vision methods employed on videos collected of pedestrians in crowded areas
Wearable Stress and Affect Detection (WESAD) dataset [32]	ACC, BVP, ECG, EMG, EDA, RESP, TEMP	Multimodal	Abnormal Behavior Detection	This dataset is a publicly available collection of physiological data from 15 individuals recorded during a lab experiment using chest- and wrist-worn devices. The data were collected under five conditions: Baseline, Amusement, Stress, Meditation, and Recovery [32]. Tphysiological data from 15 subjects captured from the wrist and chest-worn devices
Multimodal Analysis of Human Nonverbal Behavior in Conversations – Human–Computer Interaction. (MAHNOB-HCI) dataset [33]	Audio signals, face videos, eye gaze data, and peripheral/ central nervous system physiological signals	Multimodal	Abnormal Behavior Detection	This dataset comprises multimodal data, including face videos, audio signals, eye gaze data, and physiological signals from the peripheral and central nervous systems. Two experiments were conducted with 27 participants of diverse cultural backgrounds and genders. Participants watched 20 emotional movies in the first experiment and self-reported their feelings using specific emotional keywords. The second experiment involved showing short films and photos with and without tags, and participants rated their agreement or disagreement with the displayed tags. The captured movies and corresponding physical reactions were segmented and stored in a database [33]. 27 subjects watched 20 emotional videos and reported the emotions they feel
Bio-reactions and faces for emotion-based personalisation (BIRAFFE) dataset [34]	ECG, GSR, facial expression signals and hand movements through the accelerometer and gyroscope	Multimodal	Abnormal Behavior Detection	Individuals were subjected to audio–video stimuli and a three-level emotion-evoking game. The whole BIRAFFE dataset consists of data gathered from 201 out of 206 participants13. Unfortunately, some of the data was not properly collected for some participants due to, e.g., applications crashing, Bluetooth signal being lost, and poor electrode contact. Finally, the real data is available for 141 subjects [34]
MuSe (Multimodal Sentiment Analysis)-Physio dataset [35]	EDA, GSR, audio, video, heart rate, respiration	Multimodal	Abnormal Behavior Detection	In this database, human annotations were used to predict psycho-physiological responses. 69 participants (49 of them female) are aged between 18 and 39 years, providing about 6 h of data for the MuSe-Stress and MuSe-Physio sub-challenges. Besides audio, video, and texts, the participants can optionally utilise the ECG, RESP, and BPM signals Human annotations were used to predict psycho-physiological responses

### Datasets for lie/deception detection

For interpersonal, intergroup, and social functioning, deceit detection is crucial. Lying happens surprisingly frequently; the average person reports doing it once or twice daily. The deception that is misinterpreted as benign can result in financial loss, feelings of stupidity, mistrust, and even the breakdown of personal relationships [15]. False accusations of lying, on the other hand, can be embarrassing, increase interpersonal conflict, erode closeness, and end existing relationships. Hence lie/deception detection through reliable methods is very important. Numerous study paradigms and stimuli have been developed due to the intense interest in understanding lie detection. More standardized stimuli and open-access databases for deception detection research have forced academics to create experimental datasets [15].

#### Unimodal dataset

ReLiDDB (ReGIM-Lab Lie Detection Database) [17] presented a dataset containing speech signals that can be used for preliminary investigation. A study was done where pertinent acoustic parameters proved helpful in categorizing a voice signal as either false or true. The Romanian Deva Criminal Investigation Audio Recordings (RODeCAR) [71] database was developed by analyzing, processing, and conducting cross-examinations on preserved original criminal investigation recordings. It includes databases of honest and false speech. The main benefit of using the RODeCAR database is that it comprises real-life criminal investigation recordings, where the speakers are suspects or witnesses, and the conversations are unplanned and occur in the context of legal law enforcement actions. From archives, recordings totalling about 5 h have been processed. With 19 speakers (4 female, 15 male), 39% of the text is universally labelled untrue [71].

#### Multimodal datasets

The MU3D [15] dataset, created by Miami University, contains 320 videos of individuals telling lies and the truth. It is a multimodal dataset, meaning it includes multiple forms of data, such as audio and video. The purpose of the dataset is to aid in detecting deception [15]. The conversations of Eighty targets—twenty black women, twenty black men, twenty white women, and twenty white men—about their social ties were recorded in both honest and dishonest ways. Four separate videos—positive truth, positive lie, negative truth, and negative lie [15] —were produced for each target, totalling 320 videos that fully covered each target's ethnicity, sex, statement emotion, and statement truthfulness. The MU3D enables researchers to employ standardized stimuli in their studies, allowing for the improvement of lab replication, using signal detection evaluations, and promoting the consideration of racial group, gender, and potential interactions in deceit detection research.

Bag of Lies is another multimodal dataset suggested by [19], which has data from four different modalities, including audio, video, EEG, and gaze data. Thirty-five people were used in the data collection; each was shown 6–10 photos and asked to characterise them honestly or inaccurately. This resulted in 325 data points with a distribution of 163 truths and 162 occurrences of lies.

The DDPM dataset [72] contains information collected during an interview scenario where the interviewee is trying to mislead the interviewer about certain responses. The dataset includes physiological data such as heart rate and blood oxygenation level, as well as audio, RGB, near-infrared, and long-wave infrared images of the interviewee. This dataset is unique because it contains recordings from five different modalities in an interview setting and is suitable for use in remote photoplethysmography research and fraud detection [72].

In the Silesian Deception Database [16], the videos of subjects were recorded using a high-speed Basler camera [16] with a resolution of 640*480 and a frame rate of 100 frames per second. The subjects were not aware that the main focus of the research was analyzing facial expressions about deception detection to prevent the introduction of slight changes in facial expressions and blink dynamics.

A multimodal deception dataset was presented in [18]. The dataset consisted of videos of courtroom trials. The study dealt with using features of text (testimonies), audio, and video clips.

### Datasets for stress/emotion detection

Over the past century, more than 90 definitions of emotions have been proposed [78]. The understanding of emotions is complex due to the various words used to describe them and the multiple existing theories. There are different classifications of emotions, such as cognitive versus non-cognitive emotions, instinctive versus cognitive emotions, and those based on duration. Some emotions, like surprise, are short-lived, while others, like rage or love, can last for years [78]. Therefore, it is important to have databases that include a range of emotional states, and this paper surveys such datasets and categorises them as unimodal and multimodal.

#### Unimodal datasets

Emotional EEG data can be collected in a variety of ways, and various databases, including “DEAP: A Database for Emotion Analysis Using Physiological Signals” [20] and “SEED (SJTU Emotion EEG data set)” [21]. Datasets like “EEG data set for Genuine & acted emotional expressions” [52] distinguish between genuine and acted emotion. In these datasets,

- Emotions are classified as true, neutral, or fake.
- Participants in these datasets wore an EEG headset while viewing photos or movie clips on a computer monitor.
- Participants' emotions changed in response to the pictures or video clips, and these changes were seen in the EEG data.

The SJTU Emotion EEG Dataset (SEED) dataset includes the three-class emotional EEG data from 15 people [76]. The participants were shown some emotional film clips (good, negative, and neutral video clips), and the data were then collected. The participants were asked to complete a questionnaire after watching the movie as part of the data collection process to report their emotional responses for feedback. The EEG data were collected using the ESI Neuro-Scan System, which consisted of a 62-channel active AgCl electrode cap and a sampling rate of 1000 Hz that adhered to the international 10–20 system [76].

#### Multimodal datasets

A study for stress detection used the SWELL_KW dataset. It is a multimodal dataset containing readings from 25 participants subjected to neutral interruptions and pressure conditions for 3 h each [22, 46]. The data collected are computer logs, facial expressions from video recording, body postures using the Kinect 3D sensor, the ECG sensor, and the body sensors for skin conductance level. Galvanic Skin Response (GSR), heart rate (HR), and heart rate variability (HRV), both taken from ECG signals, were chosen as the main features for this investigation.

A Database for Emotion Analysis Using Physiological Signals (DEAP) [20] is a multimodal data set used to examine the affective states of people [20]. Thirty-two individuals' electroencephalograms (EEG) and peripheral physiological data were monitored while they watched 40 one-minute-long music video snippets. Each film was scored by participants based on its arousal, valence, like/dislike, dominance, and familiarity levels. Frontal face footage was also taken for 22 of the 32 participants.

PASS [23], a multimodal database of Physical Activity and stress database [23], consists of the experimental procedure employed and descriptive statistics of the neurophysiological signals captured under various circumstances. The database and the participant's answers to a questionnaire about physical and mental exhaustion have been made available to the public. Electroencephalography, heart activity, electrodermal activity, breathing data, and skin temperature are some of the modalities used here. This dataset analyzes how effective stress and physical activity are modulated simultaneously and the effects on physiological measurements and artefact production.

A dataset that uses multiple sensors to gather physiological stress signals from nurses working in real hospital environments during the COVID-19 outbreak was developed to monitor stress levels in nurses [24]. This dataset provides physiological stress indicators for nurses working in real hospital environments during the COVID-19 pandemic. It was created primarily to conduct research on stress in the workplace setting and was collected using data streams from Empatica E4 devices. Nursing is stressful, and previous research has found several stress-related characteristics. This research was conducted during increased COVID-19 cases, and the nurses faced a high volume of patients, making it a highly stressful environment. Nursing stress can be more thoroughly analyzed using wearable data and end-of-shift surveys. Wearable biometric nursing stress datasets are being created to fully understand and improve the emotional well-being of nurses in a real-world setting. These datasets will assist in developing algorithms capable of identifying work-related stress early on [24].

A benchmark data set was introduced on how much the head moves during the measurement [25]. Over the past five years, non-contact picture photoplethysmography has attracted much interest. There are several approaches for estimating the human heart rate from video sequences of the face under ambient light. The pulse rate can be useful information on a mobile service robot that encourages older people to engage in physical activity [25]. Implementing a typical processing pipeline on a mobile robot allowed for a thorough comparison of face segmentation techniques, essential for reliable pulse rate extraction even while the subject is moving.

The COHFACE [26] dataset includes 160 movies and physiological information collected from 40 healthy adults over several days. The participants in the study had an average age of 35.6 years, with a standard deviation of 11.47. The group was composed of 70% men and 30% women. Participants were recorded for one minute using a standard webcam, while their physiological data were recorded using a Blood-Volume Pulse sensor and a respiration belt. The data was synchronized and recorded using the BioGraph Infiniti Software suite. The dataset includes physiological data and video streams at a resolution of 640 × 480 pixels with 20 frames per second [26].

The MAUS [27] dataset includes data collected from 22 healthy graduate students who were given guidelines and signed a consent form before the test. The study participants' age had an average of 23 years and a standard deviation of 1.7. Participants were seated at a table and were given a task on a computer monitor while their physiological data were recorded using ECG, GSR, and PPG sensors. The data was collected using Procomp Infiniti at a sampling rate of 256 Hz. PPG was recorded using a wrist-mounted sensor at a sampling rate of 100 Hz, which was transmitted to a tablet via Bluetooth [27].

The heart rate (HR) is a vital physiological marker that indicates people's mental and emotional activity. Traditional HR measures primarily depend on contact monitors, which are uncomfortable for individuals and inconvenient. Methods for remote HR estimation from face videos have recently been proposed. Large-scale databases are needed that should allow deep representation learning methods in remote HR estimation. The Visual Information Processing and Learning-Heart Rate (VIPL-HR) database [28] is one such large-scale database that has 2,378 visible light movies (VIS) and 752 near-infrared (NIR) videos of 107 participants. Along with these additional variables, the VIPL-HR database also includes adjustments to the acquisition equipment, lighting, and head movements [28].

The Multimodal Stressed Emotion (MuSE) [29] is a multimodal dataset of stressed emotions that consists of recordings of 28 college students from the University of Michigan, nine female and 19 male, in two sessions: one during which an external stressor (the University of Michigan's final exam period) was present, and the other session during which the stressor was absent. Each recording lasts about 45 min in total. Each individual is exposed to various emotional stimuli, including brief movies and questions that evoke strong emotions. To counteract the effect of repetition while still capturing the same emotional characteristics, these stimuli are different for each session. To establish a baseline, a brief segment of the user in their natural position without any external stimuli was captured at the beginning of each session [29]. There are four basic recording techniques used to capture their behaviour: 1) A video camera, both wide-angle to catch the upper body and close-up on the face; 2) a thermal camera, close-up on the face; 3) a lapel microphone; and 4) physiological measurements, where it was chosen to measure skin conductance, skin temperature, heart rate, and breathing rate. The information includes emotional and stress self-reports (based on the PSS, or Perceived Stress Scale), as well as emotional annotations from Amazon Mechanical Turk (AMT) [29].

### Datasets for abnormal behavior detection

The timing and location associated with the sequence of activities are essential for recording an individual's daily routines. This data type can provide valuable insights into daily patterns and, as a result, detect deviations from them [83]. The most powerful signs of cognitive decline may not always be identified by observing an individual's performance at a single point but by monitoring the overall pattern and change fluctuation over time. Datasets having such patterns of activities are fundamental to detecting abnormal behaviour. We have discussed datasets that can prove noteworthy in detecting abnormality in behaviour.

#### Unimodal dataset

NLP provides a viable solution to extract concepts related to mental health used in cognitive behavioral therapy. One such platform is provided by the Koko website, where users can anonymously post about their mental health issues. The corpus contains 500,000 written posts. The corpus was annotated into three classes, thinking errors, emotions, and situations [30].

- Thinking Errors—Such errors, also known as cognitive distortions, are irrational and excessive patterns of thinking that can perpetuate mental and emotional issues, such as drawing negative conclusions, overlooking positive aspects, placing blame, and overgeneralising.
- Emotions—In CBT, emotions are frequently separated into positive and negative, beneficial/healthy and detrimental/unhealthy emotions. Here, they mostly concentrated on negative emotions pertinent to those experiencing psychological distress. A few examples of these emotions, which are considered, are shame, anxiety, guilt, jealousy, anger, etc.
- Situations. While cognitive errors and emotions were the main focus, a small set of situations, like work, health, relationship, etc., were also identified.

Multiple thinking errors, emotions, or situations could relate to a single problem [30].

#### Multimodal datasets

The WESAD [32] dataset is a publicly available collection of physiological data from 15 individuals recorded during a lab experiment using chest- and wrist-worn devices. The data were collected under five conditions: Baseline, Amusement, Stress, Meditation, and Recovery. The RespiBAN device provided data such as Electrocardiogram (ECG), electrodermal activity (EDA), electromyogram (EMG), respiration (RESP), body temperature (TEMP) at a sampling rate of 700 samples per second, and the Empatica E4 device provided data such as body temperature (4 Hz), blood volume pulse (BVP, 64 Hz), electrodermal activity (EDA, 4 Hz), and three-axis acceleration (32 Hz). This dataset is designed for research on wearable stress and effects detection.

MAHNOB-HCI [33] is a multimodal database created to research implicit tagging and emotion recognition. A multimodal setup was created to collect face videos, audio signals, eye gaze data, and physiological signals from the peripheral and central nervous systems. Two experiments involved 27 people from various cultural backgrounds and both genders. In the first experiment, participants viewed 20 emotional movies and then self-reported their feelings using emotional keywords, excitement, intensity, supremacy, and predictability. In the second trial, short films and photos were shown without any tags before being shown with either correct or erroneous tags. The participants graded how well they agreed or disagreed with the displayed tags. The physical reactions and captured movies were divided into segments and entered into a database [33].

A dataset of bio-reactions and faces for emotion-based personalisation (BIRAFFE) [34] was introduced. The experiment in emotional computing that was undertaken at the beginning of 2019 produced the BIRAFFE data set, which was then released. The experiment is a component of creating computer models for emotion recognition and classification. There was a strong conviction that such models should be designed to be individualised because each person's emotional reactions will vary depending on their personality. The International Affective Digitized Sounds (IADS) [34] and International Affective Picture System (IAPS) [34] databases, respectively, served as the sources for the auditory and visual stimuli used in the experiment.

Additionally, two paradigms were blended. The first experiment involved exposing people to stimuli and recording their physiological responses (ECG, GSR, and facial expression) later. The same reactions were recorded in the second as the individuals played simple computer games [34].

The University of Minnesota (UMN) has collected normal and abnormal crowd recordings for the Detection of Abnormal Crowd Behaviour Using the Social Force Model. The collection consists of films from 11 different escape event scenarios shot in 3 indoor and outdoor settings. Each video starts with a segment on regular behaviour and concludes with segments on deviant conduct [31].

The study conducted four sub-challenges, MuSe-Wilder, MuSe-Stress, MuSe-Sent, and MuSe-Physio [35], which focused on continuous emotion prediction, recognising five classes for valence and arousal, and predicting a novel aspect of "physiological-emotion." Two datasets were used for these sub-challenges: the Multimodal Sentiment Analysis in Car Reviews data (MuSe-CaR) [35] for the MuSe-Wilder and MuSeSent sub-challenges, and a subset of the innovative audio-visual-text Ulm-Trier Social Stress dataset (Ulm-TSST) [35] for the MuSe-Stress and MuSePhysio sub-challenges. The MuSe-CaR data is the largest emotion-annotated multimodal dataset collected in the wild. In contrast, the Ulm-TSST dataset contains 110 people highly annotated by self-reported and continuous dimensional assessments of mood [35].

The Ulm-TSST [35] dataset includes four biological signals (EDA, Respiration, ECG, and heart rate) and audio, video, and textual components. With a held-back labelled test set, these datasets provide a consistent testing environment that allows for investigating different modalities and applying advanced models in a controlled and comparable setting [35].

## Methodology

The section below reviews the methodologies used to analyse cognitive behaviour using machine learning techniques. The comparative analysis of such cognitive behaviour analysis techniques across different data modalities is presented in Table 2. Similar to datasets, the methods are divided into Lie/Deception detection, Stress/Emotion Detection, and Abnormal Behavior detection. The subsections are further divided into frameworks for unimodal and multimodal data channels. The focus is to discuss feature extraction for unimodal and multimodal and fusion methods for multimodal data.

**Table 2:** Comparative Analysis of Cognitive Behavior Analysis Techniques Across Data Modalities

Cognitive Behaviour Analysis Task	Data Modalities	Dataset Type	Feature Extraction	Description
Lie/Deception Detection	Audio	Unimodal	Mel Frequency Cepstral Coefficient (MFCC) [17] Spectral Kurtosis, MFCC, Spectral Spread, blood pressure, Spectral Centroid, respiration rate, and Tonal Power Ratio [66]	The Linear Kernel Support Vector Machines (SVM) classifier was used on the processed speech signals. The accuracy of Lie and Truth deception detection of speech audio, respectively, was 88.23% and 84.52% [17]. The MMO-DBN [66] method combines the Monarch Butterfly Optimization [95] and Moth Search [91] algorithms with a Deep Belief Neural Network, resulting in an accuracy of 98.4%
Lie/Deception Detection	Images	Unimodal	Facial Features extracted using OpenFace[39]	A fraudulent detection framework to identify persons acting dishonestly in video clips by extracting the proportions of their facial micro-expressions [38]. An expression database with five expressions (Happiness, Joy, Surprise, Anger, Disgust/Contempt, and Sadness) with a classification accuracy rate of 85% Long Short-Term Memory Network (LSTM) was trained using facial videos from Real-life Trial (RLT) Dataset, Silesian Deception Dataset, and Bag-of-lies dataset to classify facial features with an accuracy of 89.49% [39]
Lie/Deception Detection	Audio and Video	Multimodal	Verbal features: unigrams and bigrams derived from bag-of-words representation [18] Non-verbal features: Eyes, eyebrows, and mouth movements (facial expressions) and hand movements and trajectories (hand gestures)	The decision Trees algorithm was trained on these features to classify truth and deception with an accuracy of up to 75%
Lie/Deception Detection	Audio, video, and text	Multimodal	Improved Dense Trajectory (videos), MFCC (Mel-frequency Cepstral Coefficients) features from audio and GloVe vector representations for transcripts (text)	Linear SVM algorithm was applied to classify truth and deception with an accuracy of 87.73%
Lie/Deception Detection	Audio, video, and EEG	Multimodal	Attention-enhanced frequency distributed spectrograms (audio), two-stream CNN (video frames), Bi-LSTM (EEG)	The study investigates the Bag of Lies dataset using audio, video, and EEG data, applying late fusion of a two-stream CNN, attention-enhanced frequency distributed spectrograms with CNN, and a Bi-LSTM neural network for EEG data to detect lies, achieving an 83.5% accuracy with multimodal fusion
Lie/Deception Detection	Audio, video, and EEG	Multimodal	Audio frames, Concatenated LBP face images from 20 frames per video, Concatenated EEG channels	In [40], LieNet, a unique deep convolutional neural network, is developed to detect multiscale variations of dishonesty using preprocessed audio, video, and EEG signals individually input into LieNet[40] for feature extraction. The framework is trained with data augmentation methods resulting in high accuracy rates on the BOL, RL trail, and MU3D databases. Other Deception detection techniques are also reported in literature [41, 42]
Lie/Deception Detection	Audio, video, and micro-expression features	Multimodal	3D-CNN [43] (videos), CNN and Word2Vec (text), open smile [44] toolkit (audio), 39 manually annotated microexpressions	[43] proposes a neural network model for deceit detection using audio, video, text, and micro-expression features; features are extracted using 3D-CNN, CNN, openSMILE toolkit, and binary annotations; the features are fused and fed to a multilayer perceptron for classification, achieving a maximum accuracy of 96.14%
Lie/Deception Detection	Audio, Video, EEG, Gaze	Multimodal	LBP features from 20 frames per video, Zero crossing rate (audio), Spectral centroid (audio), Spectral bandwidth (audio), Spectral roll-off (audio), Chroma frequencies (audio), MFCC (audio), PyGaze (gaze), 100 points from a CSV file for each channel (EEG)	The research presented by [19] collected data from four different modalities and used different ML models to analyse and classify them, including using LBP and algorithms like SVM, random forest, and MLP for video data, frequency-based properties and Random Forest/KNN for audio data, CNN-based classifier and Random Forest/MLP for EEG data, and fixations, eye blinks and pupil size as features for gaze data
Stress/Emotion Detection	EEG	Unimodal	Differential Entropy (DE), Power Spectral Density (PSD), Differential Asymmetry (DASM), Differential Caudality, and Rational Asymmetry (RASM)	In [21], DBNs were used to classify positive, neutral, and negative emotions from EEG data filtered by a bandpass filter between 0.3 and 50 Hz, using features such as Differential Entropy (DE), Power Spectral Density (PSD), Differential Asymmetry (DASM), Differential Caudality, and Rational Asymmetry (RASM), achieving an average accuracy of 86.08%, with SVM, LR, and KNN also used as classifiers
Stress/Emotion Detection	EEG	Unimodal	empirical mode decomposition (EMD), discrete wavelet transformations (DWT) and a combination of both DWT-EMD	In [52], EEG characteristics are extracted using EMD, DWT, and DWT-EMD, and classification techniques such as KNN, SVM, and ANN were used to classify intrinsic properties of real, neutral, and performed smiles with an average accuracy of 94.3% and 84.1% using DWT-EMD and ANN in alpha and beta bands, respectively
Stress/Emotion Detection	ECG	Unimodal	Peak detection followed by HRV feature extraction	In MAUS Dataset [27], HRV statistical and frequency domain features are extracted. SVM is applied for binary classification achieving an accuracy of 71.6% for the wrist using LOSO and mixed subject fivefold cross-validation methods
Stress/Emotion Detection	PPG (Wrist)	Unimodal	Peak detection followed by HRV feature extraction	In MAUS Dataset [27], HRV statistical and frequency domain features are extracted. SVM is applied for binary classification achieving an accuracy of 66.7% wrist PPG using LOSO and mixed subject fivefold cross-validation methods
Stress/Emotion Detection	PPG (Fingertip)	Unimodal	Peak detection followed by HRV feature extraction	In MAUS Dataset [27], HRV statistical and frequency domain features are extracted. SVM is applied for binary classification achieving an accuracy of 59.9% for fingertip PPG using LOSO and mixed subject fivefold cross-validation methods
Stress/Emotion Detection	Text	Unimodal	GloVe embeddings	The cognitive approach to psychotherapy aims to modify negative thoughts; NLP was employed to create schemas from cognitive processes demonstrated by healthy individuals. These were then categorised into nine groups and mapped using GLoVE embeddings with KNN, SVM, and RNN classifiers
Stress/Emotion Detection	ECG, GSR	Multimodal	ECG: HRV (Statistical and Frequency), GSR: statistical	In the SWELL_KW dataset [22], stress detection was performed using ECG and GSR modalities with preprocessing and feature extraction methods. KNN and SVM algorithms were used for classification achieving 66.52% and 72.82% accuracy, respectively
Abnormal Behaviour Detection	Text	Unimodal	Bag of Words, SkipGram, GloVe	The corpus used in this study was taken from the Koko platform, which contains 500,000 posts on mental health issues. It was annotated into three classes: thinking errors (such as black-and-white thinking and catastrophising), emotions (including anger and anxiety), and situations (such as bereavement and work). The posts can have multiple labels, and different deep-learning techniques were used with word embeddings to classify them. The CNN-GloVe model achieved the highest F1 score of 57.8%
Abnormal Behaviour Detection	Images	Unimodal	Social Force Flow [31] For every pixel in every frame, the interaction force is then transferred into the image plane	The Social Force concept is used to locate abnormal behaviours in crowd footage by covering a picture in a grid of particles, projecting it using the space–time average of optical flow, and measuring the interaction forces between particles treated as persons. The method achieved 94% accuracy using the bag of words method to categorise frames as normal and abnormal
Abnormal Behaviour Detection	ECG	Unimodal	quadratic time–frequency distribution (QTFD) technique	This paper uses the quadratic time–frequency distribution (QTFD) technique to analyse EEG signals and track changes in spectral characteristics over time, extracting time–frequency characteristics for subject-dependent SVM classification of emotions using a 2D arousal-valence plane [50]
Abnormal Behaviour Detection	ECG	Unimodal	Power Spectral Density and the Burg Autoregressive model [51]	A technique proposed for emotion recognition combines dynamic functional network patterns with regional brain activations calculated using Power Spectral Density and the Burg Autoregressive model. The method achieved up to 90.3% accuracy in differentiating between true/genuine versus neutral, true/genuine versus fake, and neutral versus fake emotions [51]
Abnormal Behaviour Detection	ECG	Unimodal	DWT, EMD, and DWT-EMD	In [52], SVM, KNN, and ANN classifiers were used on EEG data to identify genuine smiles, fake/acted smiles, and neutral expressions. EEG features were extracted using three time–frequency analysis techniques at three frequency bands: DWT, EMD, and DWT-EMD. When distinguishing genuine emotional expression from a fake emotional expression using ANN, SVM, and KNN, the DWT-EMD technique yielded the highest classification accuracy in the alpha band at 94.3%, 92.4%, and 83.8%, respectively
Abnormal Behaviour Detection	ECG, EDA, EMG, BVP, Accelerometer, Respiration, and Temperature	Multimodal	Forward Selection method	In [54], Forward Selection was used for feature selection, and SMOTE was used to balance the imbalanced WESAD dataset, with non-linear algorithms like GBDT, RF, ET, and DT being used to evaluate information gain through Gini Impurity or Friedman MSE
Abnormal Behaviour Detection	ECG, EDA, EMG, BVP, Accelerometer, Respiration, and Temperature	Multimodal	PCA, Quantile Transformer, and Standard Scalar preprocessing	This study analyses bio-signals to detect stress using deep learning and machine learning on the WESAD dataset, applying PCA, Quantile Transformer, and Standard Scalar preprocessing, and using six machine learning methods for binary classification while employing Leave-one-subject-out cross-validation to avoid personalisation [55]
Abnormal Behaviour Detection	Accelerometer, EDA, Temperature	Multimodal	Features like Mean and Standard Deviation, Dynamic Range, and min and max values were extracted	A new stress tracking system is proposed based on a GRU RNN, which is useful in situations where not all modalities are reliable stress predictors. The system performs binary classification, considering only ACC, EDA, and TEMP signals with statistical parameters for feature engineering. GRU solves the vanishing gradient problem of RNN, and the selected indicators are used to distinguish between stress and non-stress-related circumstances [57]

### Lie/deception detection

#### Unimodal framework for lie/deception detection using speech signals

ReLiDDB [17] (ReGIM-Lab Lie Detection DataBase) uses the Mel Frequency Cepstral Coefficient (MFCC), which is frequently used in automatic speech processing for both the cases of lie detection and individual voice identification. The Linear Kernel Support Vector Machines (SVM) classifier was used on the processed speech signals. The accuracy of Lie and Truth deception detection of speech audio was 88.23% and 84.52%.

The MMO-DBN [66] method, which combines the Monarch Butterfly Optimization [95] and Moth Search [91] algorithms with a Deep Belief Neural Network, is proposed in [66] for speech signal deception detection. The input speech signals are cleaned of noise. Then various features such as Spectral Kurtosis, MFCC, Spectral Spread, blood pressure, Spectral Centroid, respiration rate, and Tonal Power Ratio are extracted. The MMO technique is then used to classify these features using a DBN, resulting in an accuracy of 98.4%.

#### Unimodal framework for lie/deception detection using facial expressions

Facial expressions emphasise two major expressions- Macro-expressions and Micro-expressions [36]. Macro expressions, such as anger, fear, happiness, sadness, etc., are much easier to understand, mainly staying between 0.5 and 5 s. Micro expressions can be described as brief, spontaneous bodily movement expressions that could be signs of dishonest behaviour [9]. Micro-expressions generally happen unconsciously and reveal a normally repressed or concealed emotion lasting less than 0.5 s. It is comparatively easier to classify macro expressions, whereas the untrained eye often overlooks micro expressions. Computer vision-based techniques are crucial for stealth systems. Early studies [37] organized human activity in movies into three different behaviour states by tracking head and hand gestures using blob analysis. A fraudulent detection framework to identify people acting dishonestly in video clips by extracting the proportions of their facial micro-expressions [38]. The steps involved in classification are as follows:

- The subject's interview is recorded using an Embedded Vision System (EVS).
- The process begins by breaking down the video into individual frames. These frames are then processed in four consecutive stages using a Lab VIEW application. The first two steps involve colour filtration and colour conversion.
- Dynamic templates are used that are geometrically based on each frame to determine the main characteristics of the facial structure.
- The features required to recognise micro expressions on the face and decide whether or not the subject is lying are extracted.
- The IMAQ vision system, integrated with NI LabVIEWTM (National Instruments, TX, USA), was used to program the detection algorithm.
- An expression database with five expressions (Happiness, Joy, Surprise, Anger, Disgust/Contempt, and Sadness) has an accuracy rate of 85%.

**Fig. 3:**
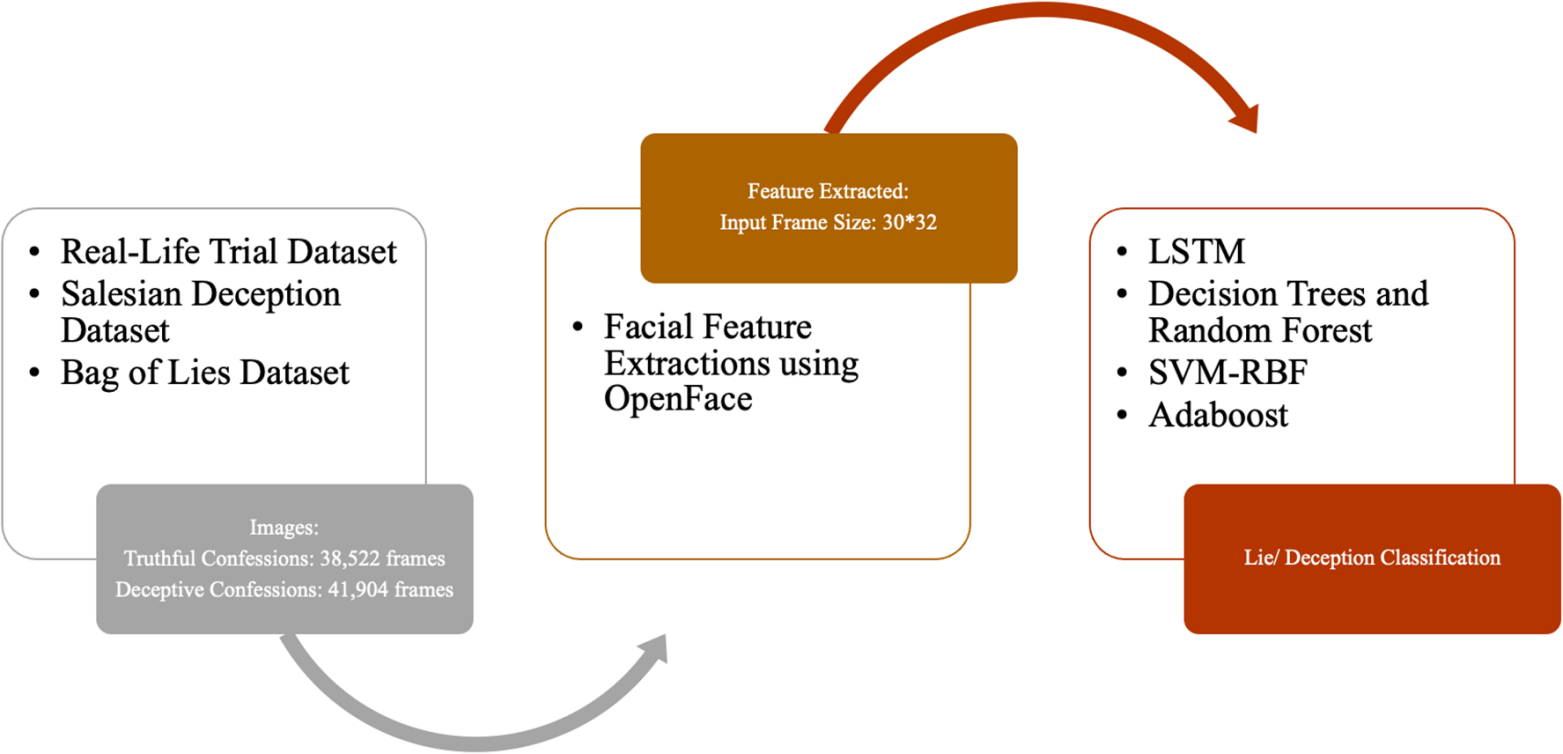
Facial Expression based Action Coding System for Detecting Deception

Long Short-Term Memory Network (LSTM) was trained using facial videos from Real-life Trial (RLT) Dataset, Silesian Deception Dataset, and Bag-of-lies dataset to classify facial features with an accuracy of 89.49% [39]. Frames of 30*30 were extracted from videos from all three datasets, and CNN was applied as a feature extractor which was then provided as input to LSTM. Figure 3 shows the classification pipeline used on the facial expressions extracted from the videos.

Other algorithms employed were:

- Adaboost with an accuracy of 88% [73]
- SVM—Radial Basis Function with an accuracy of 76.84% [74]
- Decision Trees and Random Forests with an accuracy of 75.20% [18]

#### Multimodal framework for lie/deception detection using audio and video

**Fig. 4:**
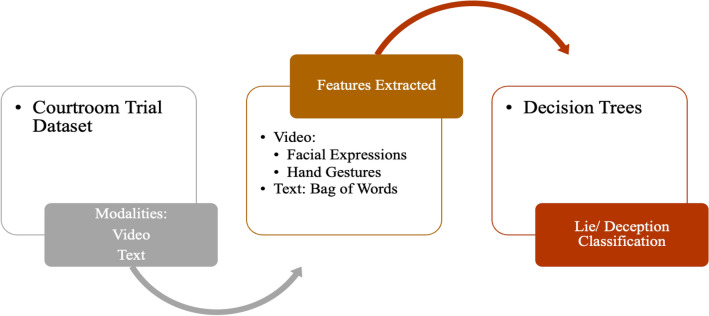
Multimodal Deception Detection using Audio and Video

Videos naturally include two types of data: audio and visual modes. The Courtroom Trial dataset [18] consisted of videos of courtroom trials. The study used features of modalities- Audio, Video, and Text as shown in Fig. 4. Through these modalities, the following features were extracted:

- Verbal features: unigrams and bigrams derived from bag-of-words representation.
- Non-verbal features: Eyes, eyebrows, mouth movements (facial expressions), hand movements and trajectories (hand gestures) were considered.

The decision Trees algorithm was trained on these features to classify truth and deception with an accuracy of up to 75%. An experiment was also conducted where a few test subjects (annotators) were asked to label truthful and deceptive convicts by reading transcripts (text), listening to audio recordings of the trial (audio), and watching muted video clips (observing expressions in silent videos) and full video clips with audio. The algorithm outperformed human labelling by a relative improvement of up to 51% [18].

Using the Miami University Deception Detection (MU3D) [15], Real-Life Trial [18], and Bag of Lies [19] datasets, the results of the multimodal framework and unimodal framework for lie detection were compared in [39]. A state-of-the-art deep convolutional neural network architecture is proposed for accurately detecting multiscale variations of deception on three multimodal datasets.

#### Multimodal framework for lie/deception detection using audio, video, and text

**Fig. 5:**
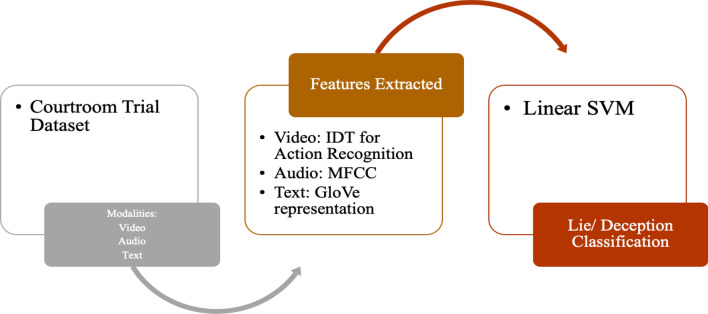
Multimodal Deception Detection using Video (Micro expressions), Audio, and Text

In Courtroom Trials Dataset [18], micro expressions are used as features from videos. Improved Dense Trajectory, a method used for action recognition, was used as a feature extraction technique for videos. In addition, MFCC (Mel-frequency Cepstral Coefficients) features from audio and GloVe vector representations for transcripts (text) were fused, as shown in Fig. 5. Linear SVM gave the highest accuracy for deception detection at 87.73% [18].

#### Multimodal framework for lie/deception detection using audio, video, and EEG

In [63], the authors investigated audio, video, and EEG data from the Bag of Lies (BoL) dataset using. Late fusion. To track facial movements from videos, rich optical flow information is extracted from a series of video frames. A two-stream CNN uses these visual cues to classify truth and lies. A speech-based deception identification system extracts attention-enhanced frequency-distributed spectrograms from audio signals. To understand variations in the frequency distribution of speech, CNN is applied. A bidirectional long short-term (Bi-LSTM) neural network is applied for the encoding and classification of EEG data to detect lies using EEG signals. Time series data represent EEG signals, and a bi-directional LSTM is used to understand the correspondence between past and future signals. The study uses the top-performing classifier to perform multimodal fusion on all modalities for lie detection. The algorithm distinguishes between dishonest and real samples with an accuracy of 83.5% when all modalities are combined.

**Fig. 6:**
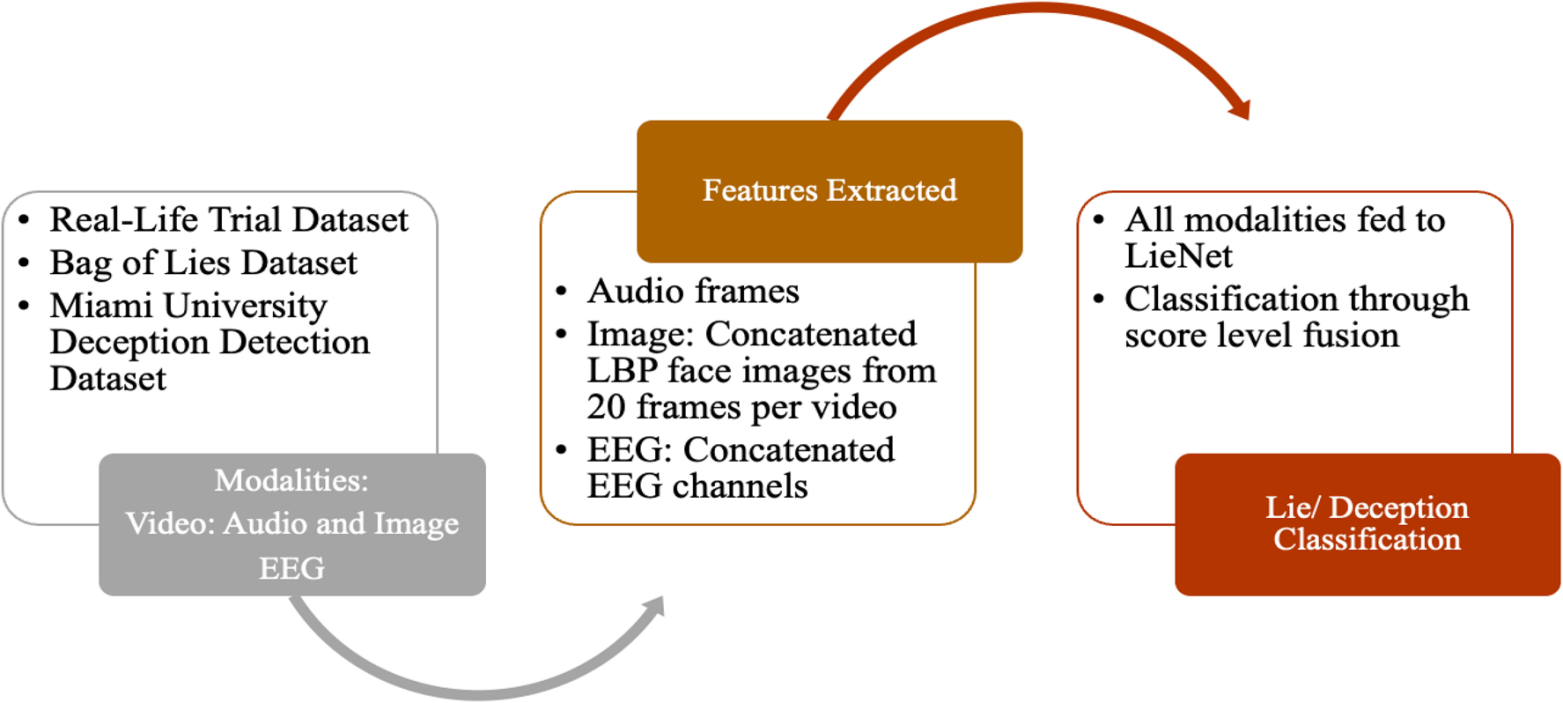
Deception Detection using Audio, Video and EEG with the LieNet model

In [40], a unique deep convolutional neural network (DCNN) called LieNet [40] is developed to detect the multistate variations of dishonesty. As shown in Fig. 6, the audio, video, and EEG signals are preprocessed as follows:

- The videos are analysed by selecting a representative frame from chunks of the video that are 20 s long. The focus is on the mouth, eyes, and nose parts, so these parts of the face are clipped from the selected frames. These images are then resized to 256*256 pixels using bilinear interpolation, and the texture is measured using Linear Binary Pattern (LBP). The LBP images are combined to form a single image of 256*5120 pixels, which is augmented and input into LieNet.
- The audio signals extracted from the videos are converted into a 2-D image using bilinear interpolation and resized to 256*256 pixels, fed into LieNet for feature extraction.
- For the Bag of Lies [19] dataset, 13 channels of EEG signals are taken into account, plotted into 256,256 images, and combined to create a single 256*3328 image. The concatenated image is then resized to 256*1024 using bilinear interpolation and input into LieNet for feature extraction.

**Fig. 7:**
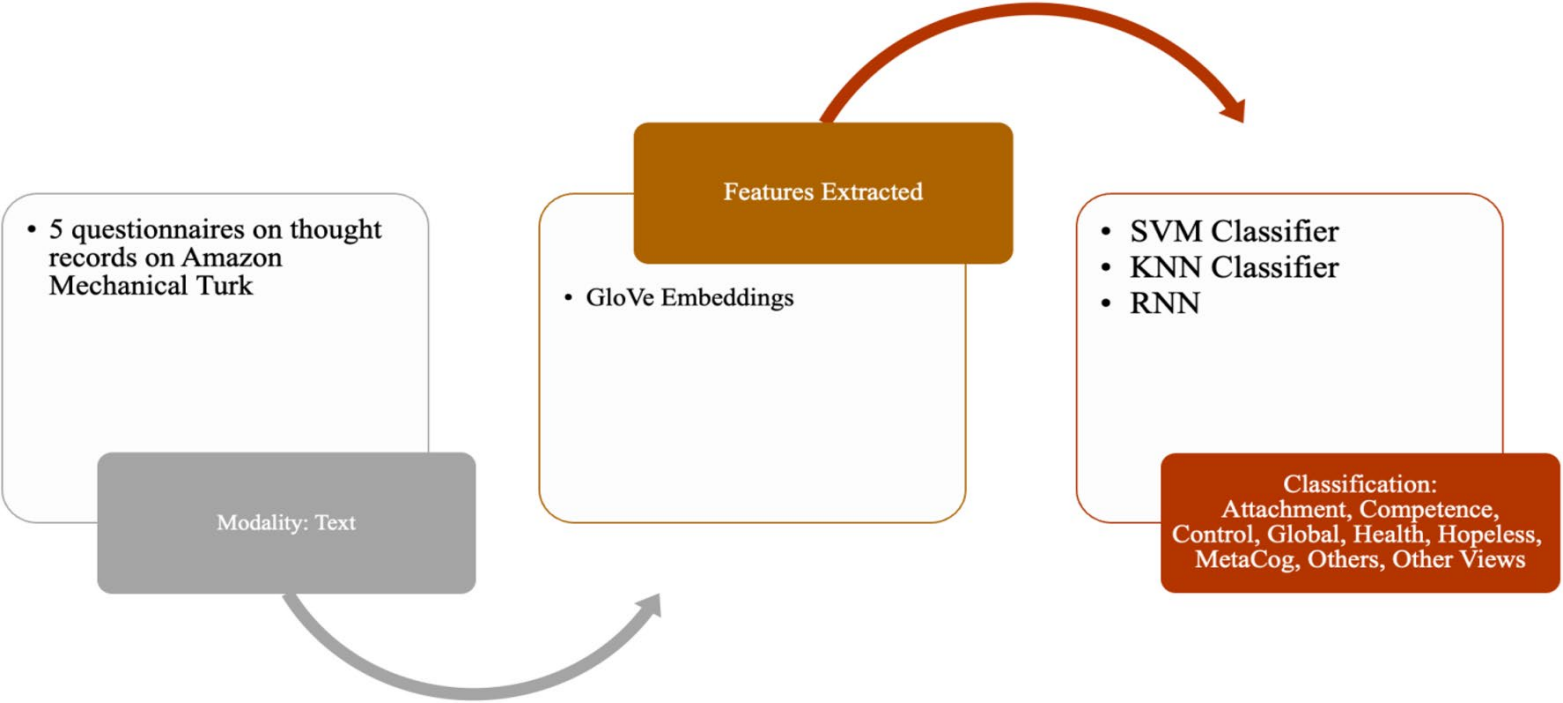
Unimodal Emotion Detection Using Text

To train the LieNet framework, data augmentation methods such as horizontal flipping, Gaussian blurring, contrast normalisation, adding Gaussian noise, and multiplying pixel values by random values between 0.2 and 0.8 are taken into consideration. Augmentation is performed on all three modalities. The LieNet framework had nine convolution layers, four max-pooling, and two fully connected layers. The activation function used here is ReLU. LieNet is applied individually on each modality and later fused at the score level to classify deception and truth. This pipeline is used separately for each dataset. (Fig. 7) The average accuracies for sets A and B of the BOL [19] dataset are 95.91% and 96.04%, respectively. On the RL trail [18] and MU3D [15] databases, LieNet's accuracy rates are 97% and 98%, respectively.

#### Multimodal framework for lie/deception detection using audio, video, and micro-expression features

An advanced neural network model for deceit detection is proposed by [43]. This method combines the audio, video, text, and Micro-Expression characteristics. The model was tested using 121 video clips from courtroom proceedings, where 60 video clips contained deception. Feature extraction was performed as follows:

- 3D-CNN [43] was applied to videos. A feature vector of 300 dimensions was obtained.
- CNN extracted features from the text taken from the transcripts (text). Word2Vec representations of the transcripts were concatenated and fed as the input layer to CNN. The final feature vector obtained had a dimension of 300 for a particular transcript.
- For Audio, the openSMILE [44] toolkit is used for high-dimensional feature extraction. Here the background noise was reduced by SoX (Sound eXchange) [45] audio processing tool. Characteristics of dimension 6373 were extracted for each input audio using the IS13-ComparE openSMILE setup. A fully connected neural network is trained to obtain a final audio feature vector with a reduced dimension of 300, similar to the dimensions of video and text features.
- Thirty-nine manually annotated micro-expressions were considered, such as smile, laughter, hands up, etc. Binary features were derived from ground truth annotations. They make up a feature vector with a dimension of 39.

A simple concatenation fused the above-extracted features and Hadamard product of video, text, and audio features, followed by concatenation with micro-expression features. The fused layer is then fed to a multilayer perceptron with a hidden layer of size 1024 and ReLU activation. The output layer is a linear classifier. The models were trained twice—text data representations were kept static in one and optimised in another. The results are as follows:

- For All Features (text data static), with MLP (simple concatenation), the accuracy is 90.49%, while with MLP (Hadamard with Concatenation), the accuracy is 90.99%
- For All Features (text data optimised), with MLP (simple concatenation), the accuracy is 95.24%, while with MLP (Hadamard with Concatenation), the accuracy is 96.14%

#### Multimodal framework for lie/deception detection using audio, video, EEG, gaze

In research presented by [19], data from four modalities, audio, video, EEG, and gaze, is collected. Different standard ML models were used for analysing and classifying the data along with feature extractions, as shown in Table 3.

- For the videos, 20 frames were picked from the recordings, and a single feature vector was created by concentrating the features extracted from these frames. The image textures are then measured using Linear Binary patterns (LBP). Further classification uses algorithms like SVM, random forest, and multilayer perceptron.
- Audio data was collected by extraction from the videos. This is processed further to calculate the different frequency-based properties like spectral centroid, zero crossing rate, spectral roll-off, chroma frequencies, spectral bandwidth, and Mel frequency cepstral coefficients (MFCC) [64][64]. Two-class classification is done using Random Forest and KNN.
- The EEG data, in the form of separate CSV files for every data point, was classified using the CNN-based classifier. The other two methods used were Random Forest and Multilayer perceptron.
- The gaze data calculates the features such as fixations, eye blinks, and pupil size. Fixations refer to instances when an individual concentrates on a specific part of the screen for a prolonged period. A modified version of the PyGaze analysis library [67] is used to calculate them.
- The results were then compared, and each modality's feature importance, impact, and influence on prediction accuracy were examined. The two sets were formed where set A has 22 test subjects with EEG values, and set B has 35 subjects without EEG values.

**Table 3:** Deception Detection using Bag of Lies Dataset with Video, Audio, EEG, and GazeModalities

Bag of lies dataset	Features extracted	Classification
Video	LBP features from 20 frames per video	SVM, Random Forest Multilayer perceptron
Audio	Zero crossing rate, Spectral centroid, Spectral bandwidth, Spectra l roll-off, Chroma frequencies, and MFCC	Random Forest KNN Classifier
EEG	100 points from each channel	Random Forest, Multilayer perceptron, EEGNet
Gaze	Fixation, Blinks, Pupil Size	Random Forest Multilayer perceptron

### Stress/emotion detection

#### Unimodal framework for stress/emotion detection using EEG

In [21], deep belief networks (DBNs) [92] were used to build EEG-based emotion detection models for classifying positive, neutral, and negative emotions. The goal was to investigate important frequency bands and channels. Noise filtration of EEG data was processed with a bandpass filter between 0.3 and 50 Hz. The EEG data were separated into identically sized, 1-s epochs without overlap for each channel. This formed 3300 clean EEG epochs. Features such as Differential Entropy (DE), Power Spectral Density (PSD), Differential Asymmetry (DASM), Differential Caudality, and Rational Asymmetry (RASM) were extracted from these EEG signals. DBN, SVM, LR, and KNN had average accuracies of 86.08%, 83.99%, 82.70%, and 72.60%, respectively [21]. The DBN has two layers, and the hyperparameter here is the number of neurons in each layer ranging from [200:500] to [150:500].

In [52], several techniques are presented for classifying the intrinsic properties of real, neutral, and staged or performed smiles using EEG. EEG characteristics are retrieved at three frequency bands using time–frequency analysis techniques, viz., empirical mode decomposition (EMD), discrete wavelet transformations (DWT), and a combination of both DWT-EMD. K-nearest neighbours (KNN), support vector machine (SVM), and artificial neural network (ANN) was then used to evaluate the proposed techniques. Different brain patterns for the three emotional expressions were visible in the power spectral feature recovered by DWT, EMD, and DWT-EMD across all frequency bands. A combination of DWT-EMD and ANN distinguished genuine emotional expressions from fake ones in the alpha and beta bands with an average classification accuracy of 94.3% and 84.1%, respectively [52].

### Unimodal framework for stress detection using ECG, fingertip Photoplethysmogram (PPG), wristband PPG

In MAUS Dataset [27], HRV statistical and frequency domain features are extracted, and SVM is applied for binary classification. The steps followed for feature extraction are as follows:

- Peak Detection
  o For ECG, a bandpass filter between 3 to 45 Hz was used.
  p For PPG, a median filter with kernel size 13 was used.
- HRV Feature Extraction
  o Time-Domain features extracted from ECG and PPG: standard deviation of inter-beat intervals (IBI) (SDNN), square root of the mean squared differences between adjacent IBI (RMSSD), the standard deviation of differences between adjacent IBI (SDSD), count or percentage of successive beats lengths that differed more than 50 ms (NN50, pNN50), IBI triangular index and the triangular interpolation of IBI histogram (TriIndex, TINN)
  p Frequency-Domain features derived from ECG and PPG: Very Low frequency(VLF, 0–0.004 Hz), Low Frequency (LF, 0.04–0.15 Hz), and high frequency (HF, 0.15–0.4 Hz), total power (TF), LF/HF ratio. The LF and HF were represented as the normalised units (nLF, nHF)

For Wrist and fingertip PPG, LOSO, and mixed subjects, five-fold cross-validation methods are used to validate the classification accuracy. SVM achieved an accuracy of 71.6%, 66.7%, and 59.9%, respectively.

#### Emotion detection using text

The cognitive approach to psychotherapy aims to alter patients' dysfunctional thoughts or excessively pessimistic beliefs about the future, the world, or oneself. They keep track of their thoughts in circumstances that elicit pathologic emotional reactions to become conscious of these viewpoints [48]. NLP creates schema out of thought patterns.

Three hundred twenty healthy people were asked to complete five questionnaires on thought records on Amazon Mechanical Turk, each of which contained multiple utterances demonstrating cognitive processes. The words and utterances were manually assigned to the schemas, which helped achieve labelled data. They were depicted using GloVe embeddings for text representation, and KNN, SVM, and RNN were used to map them to schemas [48]. Nine categories, including Proficiency, Wellness, Control, and Power, were used to group the schemas. All the utterances were divided into these schemas by finding the correlation between the words and the schema category.

#### Multimodal framework for stress/emotion detection using physiological sensors -Electrocardiogram (ECG) and Galvanic Skin Response (GSR)

**Fig. 8:**
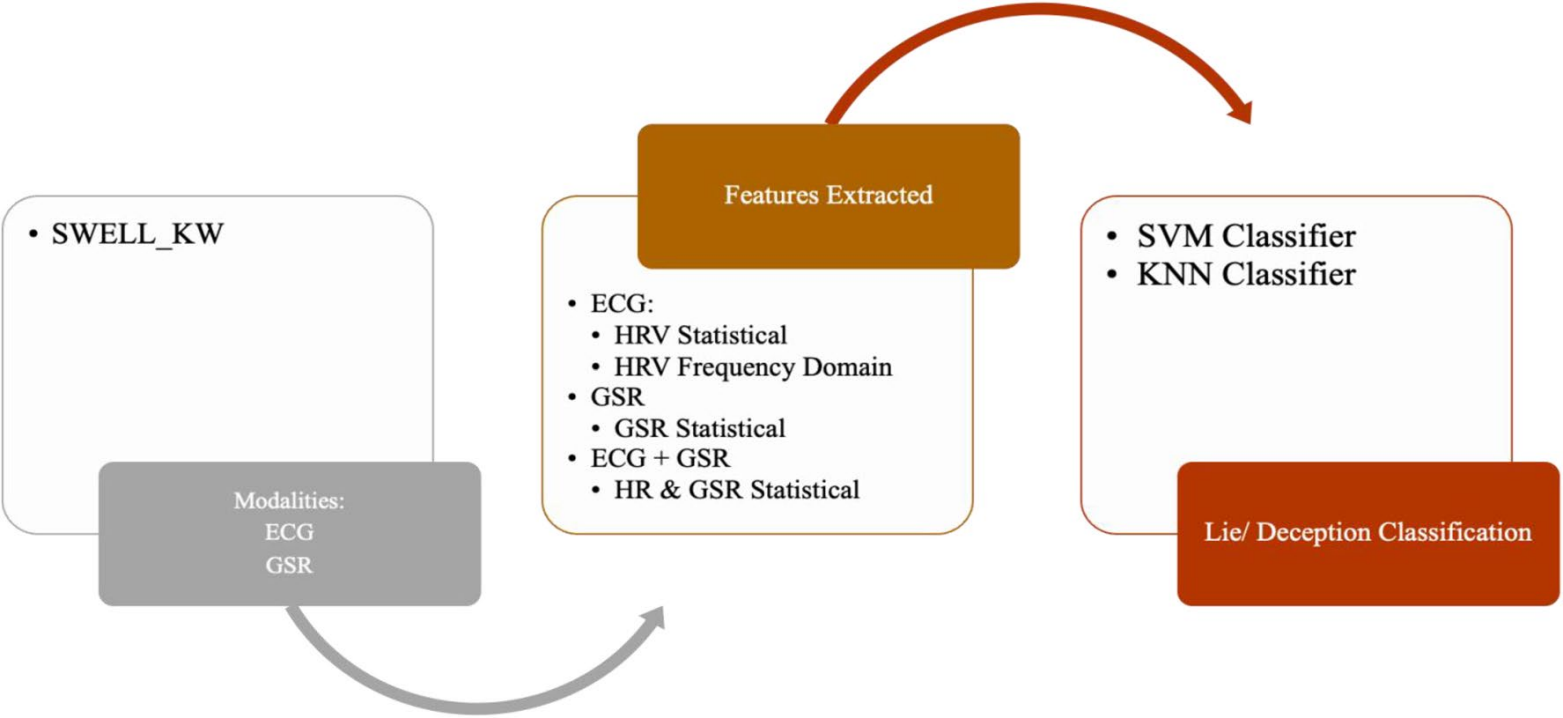
Multimodal Stress Detection Using Physiological Sensors

Stress detection was performed on the SWELL_KW dataset [22] with ECG, GSR, Body Posture, and Key Logs modalities. The use of ECG and GSR sensor data modalities is discussed here, being physiological sensors. Before using raw data from the sensors, heart rate and heart rate variability are calculated. The ECG signal is evaluated by obtaining the R-R interval and utilising the peak finder algorithm [47]. The peak counts over one minute is used to calculate the subject's heart rate. The most significant element in determining stress is the power spectral density of the Heart Rate Variability (HRV) obtained from the ECG signal extracted using the Welch method [22]. The average window approach [45] is used to preprocess the raw ECG further. GSR data is also preprocessed before features are extracted. Feature extraction is performed as depicted in Fig. 8, using the following methods:

- The mean, median, and standard deviation for both HR and GSR are considered the statistical features [22]
- HRV statistical features such as root mean square of the successive difference in distance (RMSSD), Average of NN intervals (AVNN), Standard Deviation of Average of NN intervals (SDANN), Standard Deviation of NN intervals (SDNN), Number of pairs of successive NN intervals differ by 50 ms (NN50), the ratio of NN50 to the total number of NNs (PNN50) [22]
- HRV frequency domain features are Low-frequency power from 0.04 HZ to 0.15 Hz (LF), High-frequency power from 0.15 HZ to 0.4 Hz (HF), Ratio of LF to HF (LF/HF) [22]
- GSR statistical features as mean, median, mean absolute deviation, and standard deviation [22]

A total of 17 features were extracted from ECG and GSR. KNN and SVM algorithms are used for classification. Classification accuracy of 66.52% using KNN and 72.82% using SVM was achieved.

### Abnormal behavior detection

#### Unimodal framework for abnormal behavior detection using speech signals (Audio)

Emotion could be one of the ways to understand cognitive behaviour, thus opening the door for applying AI to speech signals obtained via voice. Many deep learning techniques, including CNN, Auto Encoders, RNN, Deep Belief Networks (DBN), and Deep Boltzmann Machine (DBM), could be used. Different emotions include joy, happiness, melancholy, neutral, amazement, indifference, contempt, terror, and wrath [47]. The drawbacks of using deep learning to recognise speech emotions, including their massive internal layer-wise designs, poorer efficacy for temporally changing input data, and overlearning during layer-wise information memory, are highlighted.

#### Unimodal framework for abnormal behavior detection using text

Corpus took from the Koko platform [30], a website where users can anonymously post about their mental health issues. The corpus contains 500,000 written posts. The corpus was annotated into three classes, thinking errors, emotions, and situations [30]. Cognitive distortions, also known as thinking errors, were first described in [87] as a way of processing information that leads to predictable mistakes in thinking. The thinking errors considered in this context include black-and-white thinking, blaming, catastrophising, comparing, disqualifying the positive, emotional reasoning, fortune telling, jumping to negative conclusions, labelling, low frustration tolerance, inflexibility, mental filtering, mind-reading, over-generalising, and personalising [86]. Emotions that are studied in [30] are anger, anxiety, depression, grief, guilt, shame, jealousy, hurt, loneliness, and situations that are considered bereavement, existential, health, relationships, school/college, work, and others.

**Fig. 9:**
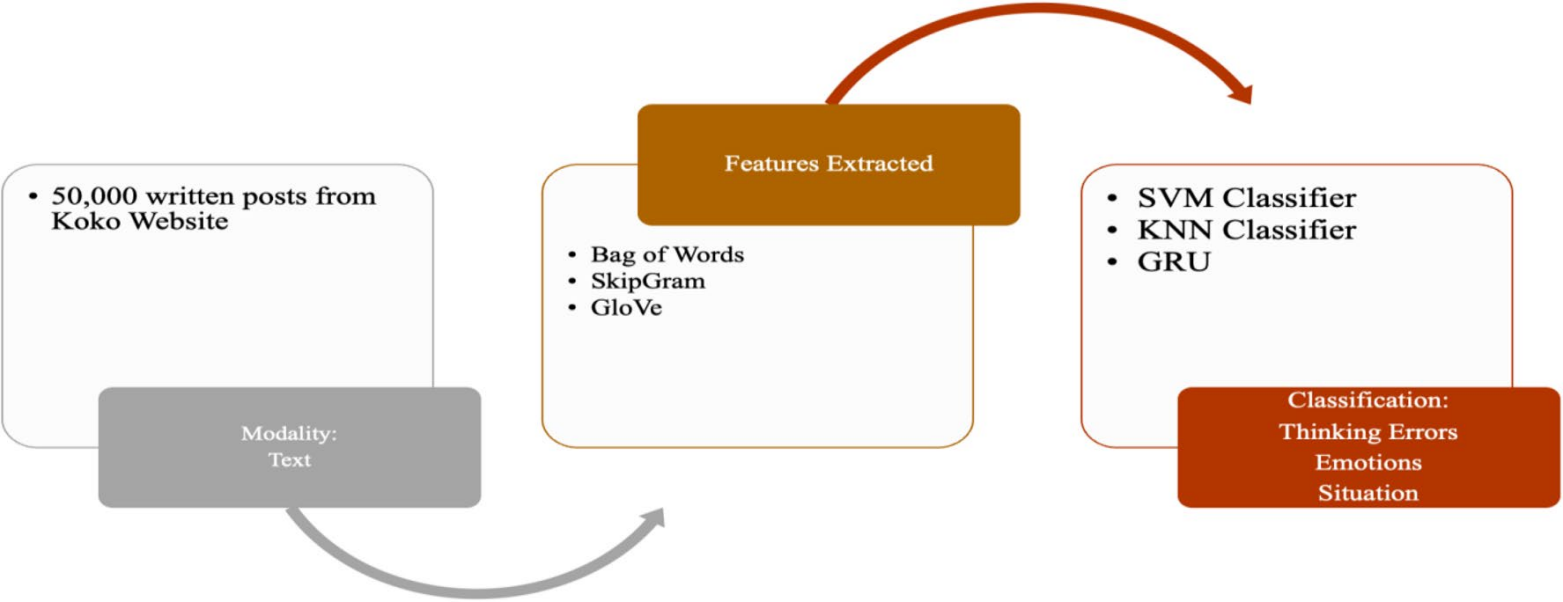
Unimodal Abnormal Behavior Detection Using Text

A single post could also be labelled with multiple thinking errors, emotions, and situations, making the process complex. Classifying the positions is similar to sentiment analysis, where detecting thinking errors and emotions are similar to negative sentiment. Since a small portion of posts is annotated, distributed representation of words has been done to obtain unsupervised learning insights. Bag-of-words, Skip-gram, and GloVe word vectors are used as an embedding mechanism, as shown in Fig. 9, with SVM, CNN, and GRU methods. CNN-GloVe gave an average F1 score of 57.8%, the highest of all classifications considered above.

#### Unimodal framework for abnormal behavior detection using images

The Social Force concept locates anomalous behaviours in crowd footage [31]. To achieve this, a picture is covered in a grid of particles and projected using the space–time average of optical flow. The social force model measures the interaction forces between the moving particles by treating them as persons. The interaction force is then transferred into the image plane to acquire Social Force Flow [31] for every pixel in every frame. Force Flow's spatiotemporal volumes are arbitrarily chosen to represent the typical crowd behaviour. Using the Bag-of-words method, the frames are categorised as normal and abnormal. Utilising interaction forces, the regions of anomalies in the aberrant frames are localised. This method achieved an accuracy of 94%.

#### Unimodal framework for abnormal behavior detection using physiological sensor—(EEG)

Automated Emotion Recognition (AER) plays a vital role in human emotion recognition. Different sensors, like contact and contactless, are used for human emotion detection [49]. A multi-model strategy is applied in this research project. EEG features can capture, compress, and then categorise emotions. The following computing stages make up the pipeline of EEG-based emotion recognition:

- EEG data are collected using 10–20 Montage systems, and ICA (Independent Component Analysis) is used to eliminate eye and muscle movements. Then, the signals are filtered using a band-pass filter, and events such as positive, negative, and neutral events are plotted in an EEG signals file. Several feature extraction methods extract emotion traits from these signals, including DWT (Discrete Wavelet Transform) and EMD (Empirical Mode Decomposition). For classification, methods like CNN, SVM, and others are employed.
- A technique known as quadratic time–frequency distribution (QTFD) [50] is utilised to analyse EEG signals in a high-resolution time–frequency space and to track changes in spectral characteristics over time. 13 time and frequency-based features are expanded to the combined time–frequency domain to measure the EEG data's time–frequency representation. Four different techniques are used to label emotions in the EEG data, using a 2D arousal-valence plane. Three standard evaluation analyses are used to measure the effects of using different EEG channel groups covering different parts of the brain. The EEG signals are transformed into Time–Frequency Representations (TFR) using a QTFD called the Choi-Williams distribution (CWD). Time–frequency characteristics are extracted from each EEG segment's CWD representation and used to classify emotions using subject-dependent SVM classifiers [50].
- A technique is proposed for emotion recognition that combines dynamics.

Functional network patterns with regional brain activations [51]. The brain activations were calculated using Power Spectral Density (PSD) and the Burg Autoregressive model. By analysing the brain activations and connection networks, distinct patterns for each of the three emotions can be observed in all frequency bands.

- In [51], the classification of three different emotions is improved by merging parameters of brain activation with patterns of functional connectivity networks, resulting in improved performance, sensitivity, specificity, and area under the receiver operating characteristic curve. The study achieved a classification accuracy of up to 90.3%, 88.52%, and 78.82% in tasks to differentiate between true/genuine versus neutral, true/genuine versus fake, and neutral versus fake emotions.
- In [52], ANN, SVM, and KNN classifiers were employed on EEG data to categorise smiles as genuine smiles, fake/acted smiles, and neutral expressions. EEG features are extracted using three time–frequency analysis techniques at three frequency bands: discrete wavelet transforms (DWT), empirical mode decomposition (EMD), and DWT-EMD. When separating genuine emotional expression from a fake emotional expression using ANN, SVM, and KNN, respectively, the DWT-EMD technique produced the highest classification accuracy in the alpha band of 94.3%, 92.4%, and 83.8%.
- In [52], SVM, KNN, and ANN classifiers were used on EEG data to identify genuine smiles, fake/acted smiles and neutral expressions. EEG features were extracted using three time–frequency analysis techniques at three frequency bands: DWT, EMD, and DWT-EMD. When distinguishing genuine emotional expression from a fake emotional expression using ANN, SVM, and KNN, the DWT-EMD technique yielded the highest classification accuracy in the alpha band at 94.3%, 92.4%, and 83.8%, respectively.
- In [53], examining several classifiers that use machine learning, including KNN, NB, SVM, and RF, SVM and RF perform better than KNN and NB for emotion recognition using EEG. With frontal lobe EEG electrodes, classification accuracy can reach over 90%, which might be the foundation for online EEG-based emotion identification.

#### Multimodal framework for abnormal behavior detection using ECG, EDA, EMG, BVP, accelerometer, respiration, and temperature

A User Independent (UI) approach was implemented on the WESAD dataset [32]. The model can be run on a new user without requiring prerequisite calibration for their affective states like baseline, amusement, and stress. There was no requirement for calibration because these UI models were trained to predict the emotionality of incoming users based on a preset set of users. The modelling was done for three different affective states: multi-affective state classification (Stress vs Amusement vs Baseline vs Meditation), Tri-affective state classification (Stress vs Amusement vs Baseline), and Bi-affective state classification (Stress vs Non-Stress) [54]. Temporal Segmentation was used as a feature extraction method. Accurate boxplots were plotted for different train-test split combinations based on which percentiles and interquartile ranges (IQR) were calculated for different affective state cases across various classifiers. The maximum, minimum, and standard deviation were determined from the collected characteristics for each portion of the sampling interval (1/700th (of a second) as 700 Hz is the sampling frequency).

**Fig. 10:**
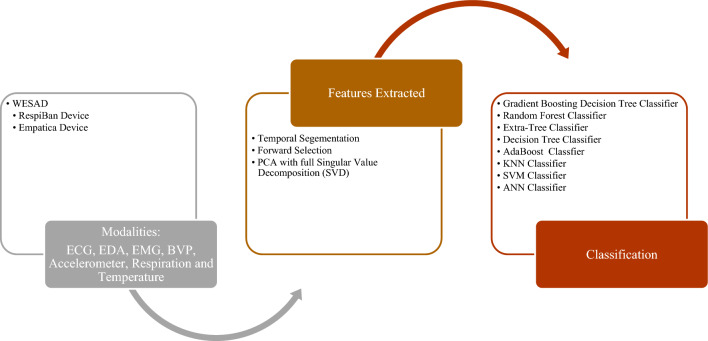
Abnormal Behaviour Detection using WESAD Dataset

Forward Selection was employed as the feature selection approach using an iterative process to select the most important features from all the features [54]. The WESAD dataset's imbalances are corrected using the oversampling technique known as Synthetic Minority Over-Sampling Technique (SMOTE), where the data distribution is as follows: 42% are in the baseline condition, 11% are having fun, 25% are meditating, and 22% are in the stressed-out state. The author has employed non-linear algorithms, as shown in Fig. 10, such as Gradient Boosting Decision Tree (GBDT), RF, Extra-Tree (ET), and DT. ET, RF, and DT used Gini Impurity to evaluate information gain, while GBDT used Friedman MSE.

Efforts are made to analyse the bio-signals to detect whether an individual is stressed using deep learning and machine learning models. The multimodal dataset, WESAD, which collects physiological sensor data from devices worn on the wrist and the chest, is the data used in this study. Principal Component Analysis (PCA) was applied with a full Singular Value Decomposition (SVD) solver and 20 components. Quantile Transformer was applied to the resultant data generated through PCA to transform the features to follow a uniform distribution [55]. Standard Scalar preprocessing was used to standardise the features by removing the mean and scaling to unit variance. Six machine learning methods, including AdaBoost (AB), KNN, RF, SVM, DT, and ANN, were employed for binary classification. The performance of these algorithms was compared. The cross-validation method Leave-one-subject-out (LOSO) was used to avoid personalisation since how people understand and react is subject-dependent.

The KNN models' performance is evaluated by hyper tuning the parameters like the total number of nearest neighbours and k-fold cross-validations parameter while classifying the WESAD dataset. The classification is done in the five different states—the baseline, stress, amusement, meditation, and transient states [56]. Using a Raspberry Pi, a KNN model was developed and trained for 30 min on every subject. Each of the 15 subjects went through this process. By hyper-tuning the cross-fold validation parameters and the number of nearest neighbours, 8 KNN models are trained and tested in this work.

#### Multimodal framework for abnormal behavior detection using accelerometer, EDA, Temperature

Based on a Gated Recurrent Unit (GRU) RNN model, a new continuous and automated mental stress tracking system has been suggested. In most circumstances, not all modalities are reliable stress predictors. Physiological parameters can change in a non-stressful situation, making it difficult to establish a standard accuracy for identifying stress; this is why utilising a GRU neural network can be useful [57]. For the binary classification, stress vs non-stress, the three signals, ACC, EDA, and TEMP, are considered. Feature engineering includes statistical parameters like Mean and Standard Deviation, Dynamic Range, and min and max values. Here, if the values are lower, the signal has regular patterns, and the measure of physiological time complexity is the sample entropy. The selected three indicators are stated to distinguish between stress- and non-stress-related circumstances.

Through the above section, we have tried to understand how to build machine learning and deep learning pipelines to classify and detect cognitive behaviours for unimodal and multimodal data. The most significant feature extraction and fusion methods for multimodal channels have been described. With the growing ease of access to powerful computational resources, efforts are being made to employ complex neural networks in this field. There is also room to explore new modalities in detecting cognitive behaviour.

## Challenges in cognitive behavioral analysis with machine learning

Some of the behavioral aspects can be satisfied by unimodal deep learning systems. At the same time, multi-factor bio and cognitive parameters contribute to behavioral analysis, so it seeks multi-modal algorithms for better performance. Multimodal applications have the potential to function in real-world circumstances. However, they pose certain challenges like missing modalities [88], lack of multimodal datasets, multimodal explainability [89], the robustness of multimodal applications [90], etc. Some of the identified challenges are detailed below.

### Unavailability of multimodal datasets

Models can become generalisable owing to the availability of multiple datasets for comparable tasks. Therefore, it is crucial to have sizable multimodal datasets in several fields. The lack of multimodal datasets for cognitive behavioral analysis makes it difficult for researchers and practitioners to train and test models that can effectively analyse and understand behaviour. Multimodal data, which includes data from multiple sources such as audio, video, and text, can provide a more complete picture of behaviour than data from a single modality. However, collecting and annotating multimodal data can be time-consuming and expensive, which may explain why currently limited options exist.

Some researchers have attempted to address this issue by creating multimodal datasets for specific tasks or domains, such as emotion recognition or mental health assessments. However, these datasets are often small and may not represent the general population. One example of a multimodal dataset that is not widely available is a dataset that contains audio, video, and text data of individuals with psychiatric disorders. At the same time, they engage in a specific task. Such a dataset would allow researchers to train models that can analyse and understand the behaviour of individuals with psychiatric disorders, aiding in diagnosing and treating these disorders. However, collecting and annotating such a dataset can be time-consuming and expensive, and it may not be widely available due to these limitations.

### Unavailability of modalities

The unavailability of modalities for cognitive behavioral analysis can limit the types of data that can be collected and analysed, which can affect the accuracy and comprehensiveness of the analysis. Behavioral data can be collected using a variety of modalities, such as audio, video, text, physiological signals, and self-report. Each modality can provide unique information about behaviour, and using multiple modalities can provide a complete picture of behaviour. However, there may be limitations regarding the availability of certain modalities for data collection. For example, some modalities, such as physiological signals, may be expensive or difficult to collect.

Additionally, some modalities may not be suitable for certain populations, such as individuals with disabilities or cultural differences. Therefore, the availability of modalities can affect the types of data that can be collected and the types of analysis that can be performed, limiting the understanding of behaviour. Some modalities could be unavailable while using the CBA application in real-time; for example, one of the sensors is not working for some time. This is termed missing modalities scenarios suffering a negative impact on accuracy and robustness [88].

### Data acquisition challenges

Data acquisition issues can make it difficult to collect and obtain the necessary data for cognitive behavioral analysis. Some of the common issues include:

- For cognitive behavioral analysis, data are collected from human activities or bio-signals from body parts. This poses challenges in data acquisition due to the subjects' freedom of mobility and improperly worn gadgets. Data privacy, annotations, participants' availability, study cost, and logistics are also challenging.
- Privacy and ethical concerns: Studies involving human participants raise ethical considerations, such as informed consent, confidentiality, and minimising harm. Behavioral data can be sensitive and personal, and obtaining consent for data collection can be challenging. One example of this would be trying to collect data on individuals diagnosed with a mental health disorder or abnormal behaviour, their consent might be difficult to obtain due to their condition, and data privacy regulations and guidelines must be followed to protect the rights of individuals.
- Limited availability of participants: Recruiting participants for behavioral studies can be time-consuming and difficult, and the availability of participants may be limited. For example, it would be difficult to find enough participants for a study of individuals with a rare disorder, resulting in a small sample size, affecting the generalizability of the results. Also, having participants across different age groups, genders, races, geographical locations, ethnicities, income levels, working professions, etc., is challenging, often limiting the study to a subset of participants.
- Real-life setting and logistical challenges: In controlled situations, stressors and motions are restricted and limited, allowing researchers to work with the subjects to ensure they correctly use the device and obtain accurate results. However, in a real-time setting, movement is unrestrained and unobserved. Additionally, the participants would like to engage in multiple activities simultaneously, which would complicate the detection process and lower the effectiveness of CBA detection systems. The participants' physiology will likely undergo significant changes due to health difficulties such as blood pressure, blood sugar, sleep patterns, drinking or smoking habits, etc. Because the concerns above could impact the system's accuracy, paying closer attention to them is imperative.
- Annotation challenges: Behavioral data often requires annotation, which can be time-consuming and resource intensive. Additionally, annotation quality may vary depending on the annotator, which can affect the accuracy of the analysis. For example, it would be difficult to find enough annotators who are experts in behavioral analysis and can provide accurate annotations. Additionally, modalities like EEG, ECG, HRV, etc., require clinical expertise to annotate and cannot be performed using normal annotation approaches such as Mechanical Turk.
- Cost: Behavioral data collection and annotation can be expensive, which may limit the availability of the data for cognitive behavioral analysis. CBA studies require longer duration and must ensure participants' enrollment, including their multiple visits. For example, collecting physiological signals such as EEG or functional magnetic resonance imaging (fMRI) can be expensive and may only be feasible for some researchers.

### Behavioral data complexity challenges

The complexity of cognitive behavioral analysis refers to the challenges associated with analysing behaviour, which can be affected by many factors. Some examples of the complexity of cognitive behavioral analysis include:

- Multimodality: Behavioral data collection from multiple modalities, such as audio, video, text, and physiological signals, is a challenge to integrate and analyse the data.
- Context-dependence: Behavior can be affected by the context in which it occurs, such as the environment, social interactions, and individual characteristics, making it difficult to generalise the findings.
- Heterogeneity: Behavior can vary across individuals and populations, making identifying patterns and generalising the findings difficult.
- Non-linearity: Behavior can be non-linear and dynamic, making it difficult to model and understand.
- Subjectivity: Behavioral data can be affected by personal perspectives and biases, making obtaining consistent and reliable results difficult.

For example, analysing the behaviour of individuals with autism can be difficult. The behaviour of individuals with autism can be affected by multiple modalities, such as physiological signals, audio, and video, making it difficult to integrate and analyse the data. Additionally, behaviour can vary across individuals with autism, making it difficult to identify patterns and generalise the findings.

### Subjectivity issues

The subjectivity issue refers to the challenge of interpreting and understanding behaviour, which can be affected by personal perspectives and biases. This can make obtaining consistent and reliable results difficult when analysing cognitive-behavioral data. Some examples of subjectivity issues include:

- Annotation bias: Behavioral data often requires annotation, and the annotations can be affected by the annotator's perspectives and biases. For example, two annotators may have different opinions about the emotion expressed in a facial expression, which can affect the accuracy of the analysis.
- Inter-rater reliability: Behavioral data often requires annotation and rating, and the reliability of the ratings can be affected by the annotator's perspectives and biases. For example, two raters may have different opinions about the severity of a patient's symptoms, which can affect the accuracy of the analysis.
- Self-report bias: Behavioral data can be collected through self-report, and the responses can be affected by the participant's perspectives, biases, and social pressures. For example, a participant may underreport or overreport their symptoms, which can affect the accuracy of the analysis.
- Interpretation bias: Behavioral data can be interpreted differently by different researchers or practitioners, and the interpretation can be affected by their perspectives and biases. For example, two researchers may have different opinions about the cause of a patient's symptoms, which can affect the accuracy of the analysis.

### Limited control over the environment

Collecting behavioral data can be logistically challenging, including multimodal data collection. For example, setting up and maintaining equipment for physiological signals and ensuring that the data is of high quality can be challenging. For example, collecting physiological signals in a naturalistic setting such as a person's home can be challenging due to the lack of control over the environment, participants' activities, the functioning of sensors, the transfer of information, etc.

Also, while testing the models for CBA, real-world environments are difficult to control, making it difficult to isolate and study specific cognitive and behavioral processes. An example of limited control over the environment in cognitive behavioral analysis is studying behaviour in a public setting, such as a mall or park. In such a setting, it cannot be easy to control for external factors that may affect behaviour, such as other people's presence and behaviour.

### Limited ability to infer causality

The limited ability to infer causality in cognitive behavioral analysis refers to the difficulty of determining the cause-and-effect relationship between different factors affecting behaviour. This can make understanding the underlying mechanisms that drive behaviour difficult, affecting the findings' accuracy and generalizability. One example of the limited ability to infer causality in cognitive behavioral analysis is studying the relationship between emotions and physiological signals. It can be difficult to infer causality between emotions and physiological signals, as physiological changes may cause emotions, or emotions may cause physiological changes. Other factors, such as cognitive or environmental factors, may also affect emotions and physiological signals, making it hard to infer causality.

### Challenges in measuring certain cognitive process

Some cognitive processes like emotions, motivation, and attention are more challenging to measure and quantify. One example of difficulty is measuring emotion regulation which refers to the ability to control and manage one's emotional experiences. It is a complex cognitive process that can be difficult to measure as it is not directly observable. Different techniques, such as self-report questionnaires, behavioral measures, and neuroimaging, can measure emotion regulation, but each has limitations and biases. There are a few more obstacles that are discussed below:

- Only seven fundamental emotions have been successfully recognised thus far. Research should be conducted to identify more than seven emotions [80].
- Electromyography (EMG) signals use muscle movement data and features like skin temperatures are still being developed for emotion identification. An extensive investigation can verify the effectiveness of these techniques.
- Accessing relevant data is a major challenge, especially in a novel paradigm where we try to detect micro-expressions through different modalities. Micro-expressions continue to be a difficult problem because they involve increasingly delicate and spontaneous facial movements that occur unconsciously.
- Human facial expressions have historically been investigated using static 2D photographs or 2D video sequences. However, a 2D-based analysis needs help managing huge variations in position and subtle facial behaviours [81].
- Caution must be taken while interpreting the results when fewer subjects are in the dataset on whom experiments were performed.

### Data pre-processing challenges

Data preprocessing and the application of AI algorithms pose another level of challenges. The most difficult aspects of creating any detection model are gathering data in a real-time setting, eliminating distortions and noise, and guaranteeing data correctness. Data pre-processing can be complex for cognitive behavioral analysis due to the nature of the data and its variability. Some common complexities include as follows:

- Data cleaning and normalisation: Behavioral data can be noisy and inconsistent, and cleaning and normalising the data can be time-consuming and challenging. For example, cleaning and normalising physiological signals such as EEG or ECG can be challenging due to artefacts and noise in the signals.
- Data alignment and synchronisation: Behavioral data can be collected from multiple modalities and sources, and aligning and synchronising the data can be difficult, especially for multimodal data. For example, aligning and synchronising video and audio data can be challenging due to frame rate variations and audio quality variations.
- Data annotation and labelling: Annotation quality may vary depending on the annotator, which can affect the accuracy of the analysis. For example, annotating facial expressions or body language can be challenging and vary depending on the annotator's expertise.
- Handling missing data: Behavioral data can have missing values, and handling missing data can be challenging and affect the analysis's accuracy. For example, missing physiological signals such as ECG or EEG data can be challenging to interpolate or fill in and may affect the overall analysis.
- Handling outliers: Behavioral data can have outliers, which can be challenging and affect the analysis's accuracy. For example, handling outliers in physiological signals such as ECG or EEG can be challenging and may require expert knowledge of the signals.

#### Unavailability of pre-trained models

The unavailability of pre-trained models for cognitive behavioral analysis can make it difficult for researchers and practitioners to quickly and easily apply machine learning techniques to analyse and understand behaviour. Pre-trained models, already trained on a large dataset and can be fine-tuned for a specific task, can save time and resources compared to training a model from scratch. However, pre-trained models for cognitive behavioral analysis are currently limited. Attempts have been made to address this issue by developing pre-trained models for specific tasks or domains, such as emotion recognition. However, these models may not be widely available or generalised to other tasks or domains. One example of a pre-trained model that is not widely available is a model that can analyse and understand the behaviour of individuals with depression. Such a model would be trained on a large dataset of physiological signals and audio and video data of individuals with depression. It could be fine-tuned for specific tasks, such as recognising patterns of behaviour characteristic of depression. However, the availability of such a model is limited due to the need for large, labelled datasets for training models and the complexity of behavioral data, which can be multimodal and context-dependent.

#### Unavailability of standards

The design and execution of the study may be inconsistent across studies, making it difficult to compare results and draw conclusions. One example of a need for more consistency in cognitive behavioral analysis is studying behaviour in different cultures. In such a setting, ensuring that the study is conducted consistently and comparably across different cultures can be challenging, as the behaviour may vary widely. This can make comparing and generalising the findings across different cultures difficult.

## Conclusion and future scope

The use of AI for cognitive behavioral analysis has the potential to provide new insights into human behaviour understanding and subsequent breakthrough user-centric applications in security, healthcare, and marketing domains. This study outlined key details regarding earlier studies, including unimodal and multimodal sensing mechanisms, feature extraction, methods utilised in AI/ML models, datasets and their benefits, drawbacks, and difficulties. AI-based cognitive behavioral analysis is a promising area of research. However, it is still in its early stages of development, and more research is needed to fully understand this technology's capabilities and limitations. Additionally, it is important to ensure that any AI-based systems are developed and used ethically, considering the potential impact on individuals and society. The future scope of cognitive behavioral analysis using AI to remove current challenges includes the following:

## Devices and modalities

- Constructing a robust, user-friendly, and flexible multimodal device with sensors (HR, HRV, and GSR) shall be considered to collect data consistently and reliably.
- Combine existing modalities, such as physiological signals and video, to create multimodal datasets and improve the quality and accuracy of existing modalities, such as EEG or ECG, to reduce noise and artefacts. This can increase the understanding of behaviour and improve the accuracy of the analysis.

## Algorithms & approaches

- Multiple measures, such as self-report, behavioral observations, and physiological measures, can provide a more comprehensive and objective view of cognitive-behavioral processes.
- Getting modality-invariant representations also requires training models to handle missing modalities. The capacity to manage missing modalities can improve models' interpretability, fairness, and robustness.
- Improving existing pre-trained models by fine-tuning them with more diverse and larger datasets, making them more robust and generalisable. Pre-trained models can be developed for different cultures and languages, which can help overcome cultural bias and language barriers in behavioral analysis.
- Deep learning can improve the effectiveness and precision of stress/emotion detection, lie/deception detection, and abnormal behaviour detection.

## Evaluation and policies

- Developing and implementing new methods for protecting behavioral data's privacy and security can help overcome data sharing and pooling concerns.
- Developing standardised protocols and computational tools for data collection, analysis, and reporting can ensure consistency across studies and participants.
- The development of concise evaluation methods and suitable diagnostic instruments must be prioritised. For each of its goals, tools should assess how well detection approaches work.

In this context, our study on cognitive behavioral analysis focused on lie/deception detection, stress/emotion, and abnormal behaviour detection will lay the foundation for further research on AI-based cognitive behaviour analysis.

## Data Availability

Sharing and pooling existing data using data sharing platforms, data repositories, data access agreements, crowdsourcing data, and interdisciplinary collaboration by collaborating with experts from different fields, such as cognitive science, computer science, and psychology. Data Augmentation by applying technical methods. Creating synthetic datasets using Generative models, such as GANs. Increasing the diversity of data by collecting data from different cultures, age groups, and populations. Databases with 3D facial expressions should be considered to evaluate better the tiny structural changes intrinsic to spontaneous emotions [81].
